# Subsurface biogeochemical cycling of nitrogen in the actively serpentinizing Samail Ophiolite, Oman

**DOI:** 10.3389/fmicb.2023.1139633

**Published:** 2023-04-21

**Authors:** Kaitlin R. Rempfert, Daniel B. Nothaft, Emily A. Kraus, Ciara K. Asamoto, R. Dave Evans, John R. Spear, Juerg M. Matter, Sebastian H. Kopf, Alexis S. Templeton

**Affiliations:** ^1^Department of Geological Sciences, University of Colorado, Boulder, CO, United States; ^2^Department of Civil and Environmental Engineering, Colorado School of Mines, Golden, CO, United States; ^3^School of Biological Sciences, Washington State University, Pullman, WA, United States; ^4^Quantitative Biosciences and Engineering, Colorado School of Mines, Golden, CO, United States; ^5^National Oceanography Centre, University of Southampton, Southampton, United Kingdom

**Keywords:** serpentinization, water–rock interaction, deep subsurface biosphere, nitrate, nitrogen isotopes, nitrogen, Samail Ophiolite

## Abstract

Nitrogen (N) is an essential element for life. N compounds such as ammonium (${\text{NH}}_{4}^{+}$) may act as electron donors, while nitrate (${\text{NO}}_{3}^{-}$) and nitrite (${\text{NO}}_{2}^{-}$) may serve as electron acceptors to support energy metabolism. However, little is known regarding the availability and forms of N in subsurface ecosystems, particularly in serpentinite-hosted settings where hydrogen (H_2_) generated through water–rock reactions promotes habitable conditions for microbial life. Here, we analyzed N and oxygen (O) isotope composition to investigate the source, abundance, and cycling of N species within the Samail Ophiolite of Oman. The dominant dissolved N species was dependent on the fluid type, with Mg^2+^-${\text{HCO}}_{3}^{-}$ type fluids comprised mostly of ${\text{NO}}_{3}^{-}$, and Ca^2+^-OH^−^ fluids comprised primarily of ammonia (NH_3_). We infer that fixed N is introduced to the serpentinite aquifer as ${\text{NO}}_{3}^{-}$. High concentrations of ${\text{NO}}_{3}^{-}$ (>100 μM) with a relict meteoric oxygen isotopic composition (δ^18^O ~ 22‰, Δ^17^O ~ 6‰) were observed in shallow aquifer fluids, indicative of ${\text{NO}}_{3}^{-}$ sourced from atmospheric deposition (rainwater ${\text{NO}}_{3}^{-}$: δ^18^O of 53.7‰, Δ^17^O of 16.8‰) mixed with ${\text{NO}}_{3}^{-}$ produced *in situ* through nitrification (estimated endmember δ^18^O and Δ^17^O of ~0‰). Conversely, highly reacted hyperalkaline fluids had high concentrations of NH_3_ (>100 μM) with little ${\text{NO}}_{3}^{-}$ detectable. We interpret that NH_3_ in hyperalkaline fluids is a product of ${\text{NO}}_{3}^{-}$ reduction. The proportionality of the O and N isotope fractionation (^18^ε / ^15^ε) measured in Samail Ophiolite ${\text{NO}}_{3}^{-}$ was close to unity (^18^ε / ^15^ε ~ 1), which is consistent with dissimilatory ${\text{NO}}_{3}^{-}$ reduction with a membrane-bound reductase (NarG); however, abiotic reduction processes may also be occurring. The presence of genes commonly involved in N reduction processes (*narG, napA, nrfA*) in the metagenomes of biomass sourced from aquifer fluids supports potential biological involvement in the consumption of ${\text{NO}}_{3}^{-}$. Production of ${\text{NH}}_{4}^{+}$ as the end-product of ${\text{NO}}_{3}^{-}$ reduction *via* dissimilatory nitrate reduction to ammonium (DNRA) could retain N in the subsurface and fuel nitrification in the oxygenated near surface. Elevated bioavailable N in all sampled fluids indicates that N is not likely limiting as a nutrient in serpentinites of the Samail Ophiolite.

## 1. Introduction

The terrestrial subsurface is known to host a substantial biosphere (2–6 × 10^29^ cells; 23–31 Pg carbon) of diverse microbial communities that likely play significant roles in biogeochemical cycling on a global scale (Nyyssönen et al., 2014; Magnabosco et al., 2018; Flemming and Wuertz, 2019). However, life in the continental subsurface is not uniformly distributed due to heterogeneity in energy availability resulting from differences in host rock lithology and the degree of hydrologic connectivity in the subsurface (Templeton and Caro, in press). Organic matter is scarce in hard-rock subsurface ecosystems, and thus, electron donors derived from minerals are the primary substrate for biological metabolism. Minerals can be directly dissolved by microorganisms, or energy can be released through abiotic chemical reactions (Escudero et al., 2018). For example, hydration and oxidation reactions that occur during the serpentinization of olivine and pyroxene in ultramafic rock can yield reducing power in the form of hydrogen gas (H_2_) (McCollom and Bach, 2009). Thus, H_2_ generation by serpentinization could fuel microbial life in peridotite rock, where sufficient oxidants are delivered hydrologically. Multiple studies have investigated the diversity and activity of microbial communities likely sustained by H_2_ production in serpentinite aquifers (Rempfert et al., 2017; Fones et al., 2019; Sabuda et al., 2020; Seyler et al., 2020; Kraus et al., 2021; Nothaft et al., 2021; Templeton et al., 2021). However, the origins of nutrients and oxidants for these communities have not been sufficiently investigated, and so the broader habitability of subsurface serpentinizing environments remains unconstrained. In particular, the source and principal form of nitrogen (N) in terrestrial serpentinite-hosted ecosystems is unknown.

Nitrogen is essential to all life on Earth as it is required to synthesize proteins, nucleic acids, and many biological macromolecules. Accordingly, the availability of N may control the productivity of ecosystems or the structure of microbial communities where it is limiting. N exists in multiple oxidation states and thus can be utilized by life for energy metabolism in addition to biosynthesis. Reduced nitrogen species such as ammonia/ammonium (NH_3_/${\text{NH}}_{4}^{+}$) may act as electron donors, while N-oxides such as nitrate (${\text{NO}}_{3}^{-}$) and nitrite (${\text{NO}}_{2}^{-}$) can serve as electron acceptors. N-oxides are especially important in the deep biosphere because oxidants are often scarce (Jones et al., 2018; Meyer-Dombard and Malas, 2022; Mosley et al., 2022). Determining the source and speciation of N accessible to serpentinite-hosted subsurface life is crucial for understanding how N availability may influence the microbial habitability of subsurface environments. In particular, tracing the fate of ${\text{NO}}_{3}^{-}$ could provide insight into the habitability of subsurface rock-hosted environments on other planetary bodies where ${\text{NO}}_{3}^{-}$ is likely present, such as Mars (Stern et al., 2017).

We measured the N and oxygen (O) isotopic composition (δ^15^N and δ^18^O, respectively) of dissolved ${\text{NO}}_{3}^{-}$ and the N isotopic composition of NH_3_/${\text{NH}}_{4}^{+}$ to assess the origin and transformation of N in the subsurface of a terrestrial serpentinizing system in the Samail Ophiolite, Sultanate of Oman, the world's largest massif of serpentinized peridotite rock (Nicolas et al., 2000). Groundwater fluids were collected from deep boreholes hosted within peridotite and gabbro. The reaction histories of sampled fluids were inferred by geochemical composition, and the speciation and isotopic composition of fluid N were analyzed with the goals of: (1) identifying the major sources of N in the aquifer and (2) evaluating the subsequent biogeochemical cycling of N in the subsurface. In addition, we evaluated possible geologic sources of N by measuring the δ^15^N of peridotite rock obtained from diamond drilling during Phase 2 of the Oman Drilling Project (Kelemen et al., 2020). Finally, the potential for microbial participation in the cycling of N at depth was assessed based on the presence of functional genes for N metabolisms in metagenomes derived from biomass collected from borehole fluids. This combined isotopic and functional gene approach yields new insights into the N dynamics of subsurface, serpentinite-hosted ecosystems, revealing how ${\text{NO}}_{3}^{-}$ introduced into serpentinite aquifers is primarily converted to ${\text{NH}}_{4}^{+}$, and how NH_3_/${\text{NH}}_{4}^{+}$ is recycled, retaining a substantial pool of fixed N in this subsurface habitat.

## 2. Methods

### 2.1. Sampling and geochemical characterization of fluids

We obtained subsurface fluids over four annual field seasons (2015–2018) from 12 boreholes previously drilled by the Oman Ministry of Regional Municipalities and Water Resources. These boreholes are situated in crustal gabbros and mantle peridotites in the Wadi Tayin block of the Samail Ophiolite. We additionally sampled borehole BA1A of the Oman Drilling Project multi-borehole observatory during the 2018 field season; the hydrological properties of this borehole are described extensively in Lods et al. (2020). The lithologies, geographic coordinates, elevations, depths, and casing properties of the boreholes are listed in Table 1.

**Table 1:** Lithology, geographic location, elevation, depth, and borehole properties of wells sampled as previously reported by Rempfert et al. (2017) and Nothaft et al. (2021).

**Well**	**Well depth (mbgl)**	**Casing extent (mbct)**	**Screened Interval (mbct)**	**Depth to water (mbct)**	**UTM Easting**	**UTM Northing**	**Elevation (mabsl)**	**Lithology**
BA1A	400.0	22.0	Open below casing	13.47	674492	2531354	583	peridotite
CM2A	400.0	23.7	Open below casing	13.4	636988	2534284	713	gabbro
NSHQ04	304.0	5.8	Open below casing	4.7	670971	2531699	543	peridotite
NSHQ14	304.0	5.8	Open below casing	9.2	675495	2529716	526	peridotite
WAB103	101.0	101.0	90–98	15	648577	2530362	632	gabbro
WAB104	120.4	120.4	101–104	40	643099	2541124	842	peridotite
WAB105	120.5	120.5	110–117	16.5	644678	2536524	738	peridotite
WAB188	78.0	78.0	35–51	9.5	671123	2529798	514	gabbro
WAB55	102.0	102.0	8–97	7.5	634777	2506101	531	peridotite
WAB71	136.5	136.5	128–131	8.3	670322	2533981	608	peridotite
NSHQ3B	472.0	185.0	91–180	-	645068	2536069	688	alluvium
NSHQ10	304.0	5.8	Open below casing	14.3	645706	2502793	453	peridotite
NSHQ21	233.0	5.4	Open below casing	3.33	633569	2509105	514	gabbro

Detailed descriptions of fluid sampling and aqueous geochemical analyses are reported in Rempfert et al. (2017), Kraus et al. (2021), and Nothaft et al. (2021) for the 2015–2016, 2017, and 2018 field seasons, respectively, with key geochemical parameters summarized in Supplementary Table 1. Briefly, a Grundfos SQ-85 submersible pump was used to collect subsurface fluids for isotopic and metagenomic analyses. Water temperature, pH, and oxidation-reduction potential were measured in the field with a Hach (Loveland, CO) HQ40D Portable Multi Meter. Boreholes were pumped ~20 min prior to sampling until pH stabilized. Biomass was concentrated for DNA extraction on a 0.2-μm Millipore polycarbonate filter. Two aliquots of fluid for isotopic analyses were filtered through a 0.2-μm filter to remove cells and collected in acid-washed 15-ml Falcon tubes (Corning Inc., Corning, NY) (Granger and Sigman, 2009). One aliquot was acidified to a pH of < 2 with concentrated hydrochloric acid for the analysis of δ^15^N of reduced N (N_red_) (U. S. Environmental Protection Agency, 1983); the other aliquot was left unacidified for δ^15^N and δ^18^O analyses of ${\text{NO}}_{3}^{-}$ and ${\text{NO}}_{2}^{-}$. Filters for DNA extraction were flash-frozen, transferred in a liquid nitrogen dewar, and stored at −80°C until extraction. Fluid aliquots for isotopic analyses were stored in a cooler on ice in the field, transported *via* air cargo at room temperature, and then stored frozen at −20°C until analysis (Avanzino and Kennedy, 1993; Menchyk et al., 2014).

At BA1A, a packer system (Solexperts) was deployed to sample discrete depth intervals in the borehole. A detailed description of sampling with the packer system is provided by Nothaft et al. (2021).

A single rain event was sampled in 2017 for ~1 min of rainfall by holding an open, acid-washed 15-mL Falcon tube at ~5 ft over the ground. The tube was not opened until after the rain event had started in order to minimize the potential contamination of the sample with dust. The sample was immediately filtered through a 0.2-μm polycarbonate filter to remove cells and prevent the biological processing of N and then placed on ice in a cooler in the field. The sample was stored frozen at −20 °C until the analysis of δ^15^N and δ^17^O + δ^18^O of ${\text{NO}}_{3}^{-}$. Since the sample volume was limited, no second aliquot was acidified for the measurement of reduced N compounds. Because precipitation in Oman is scarce and sporadic (Weyhenmeyer et al., 2002), this sample was the only rainwater obtained during field sampling throughout the multiyear campaign.

### 2.2. Classification of fluid reaction histories

Serpentinized fluids were categorized as Mg^2+^-${\text{HCO}}_{3}^{-}$ or Ca^2+^-OH^−^ type compositions according to pH and concentrations of ∑Mg, ∑Ca, and ∑CO_2_ (Supplementary Table 1) that reflect the extent of water–rock reaction (Barnes et al., 1967; Barnes and O'neil, 1969; Bruni et al., 2002; Paukert et al., 2012; Chavagnac et al., 2013). We infer that Mg^2+^-${\text{HCO}}_{3}^{-}$ fluids reacted in an open system with atmospheric CO_2_ over relatively short residence times, whereas Ca^2+^-OH^−^ fluids reacted extensively over long residence times at depths closed to atmospheric inputs (Paukert et al., 2012; Paukert Vankeuren et al., 2019; Leong and Shock, 2020; Leong et al., 2021). The degree of mixing between Mg^2+^-${\text{HCO}}_{3}^{-}$ and Ca^2+^-OH^−^ fluid types was estimated using ∑Si as a conservative tracer because ∑Si is far more sensitive to mixing than pH in ophiolitic groundwater (Leong et al., 2021). Using the mixing model predictions published by Leong et al. (2021) for endmember Ca^2+^-OH^−^ fluids containing 20 μmole/kg ∑CO_2_, the modeled concentration of ∑CO_2_ that most closely resembles ∑CO_2_ measured in highly reacted fluids in this study (Supplementary Table 1), we applied a linear model of ∑Si and extent of mixing (%) to our measured fluid compositions (Supplementary Table 2).

### 2.3. Analysis of aqueous N species

${\text{NO}}_{3}^{-}$ and ${\text{NO}}_{2}^{-}$ concentrations were quantified using a Griess reaction-VCl_3_ sequential colorimetric assay (García-Robledo et al., 2014) on a BioTek Synergy 2 Microplate Reader. ∑NH_3_ (NH_3_ + ${\text{NH}}_{4}^{+}$) concentrations were also quantified spectrophotometrically on a microplate reader using a salicylate hypochlorite colorimetric assay (Ruppersberg et al., 2017).

The δ^15^N and δ^18^O of ${\text{NO}}_{3}^{-}$ and ${\text{NO}}_{2}^{-}$ as well as the ^15^N composition of ∑NH_3_ were determined using the denitrifier method (Sigman et al., 2001; Weigand et al., 2016) in the Sigman Lab at Princeton University using 20 nmol ${\text{NO}}_{3}^{-}$ per analysis. Samples that exhibited >1% ${\text{NO}}_{2}^{-}$ were subjected to ${\text{NO}}_{2}^{-}$ removal through the sulfamic acid method prior to the analysis of the remaining ${\text{NO}}_{3}^{-}$ (Granger and Sigman, 2009) and analyzed in parallel with untreated aliquots (${\text{NO}}_{3}^{-}$ + ${\text{NO}}_{2}^{-}$) to allow for the inference of ${\text{NO}}_{2}^{-}$ isotopic composition by mass balance. Calibration of isotopic measurements was conducted with the IAEA-NO3 [δ^15^N = 4.7‰ vs. air, δ^18^O = 25.6‰ vs. Vienna Standard Mean Ocean Water (VSMOW)] and USGS34 (δ^15^N = −1.8‰ vs. air, δ^18^O = −27.9‰ vs. VSMOW) potassium nitrate standards at two concentrations (to correct for volumetric effects) every eight samples with analytical precision: 0.1‰ for δ^15^N and 0.3‰ for δ^18^O (1σ, *n* = 122). Prior to analysis with the denitrifier method, ∑NH_3_ was oxidized to ${\text{NO}}_{3}^{-}$ *via* the persulfate method using N-clean recrystallized potassium persulfate (Wang et al., 2015). These measurements are reported as δ^15^N of N_red_ because persulfate oxidizes all reduced N in the sample. An additional suite of amino acid isotope standards was used to correct for ${\text{NO}}_{3}^{-}$ contamination of persulfate (USGS 40, δ^15^N = −4.5‰; and USGS 41, δ^15^N = 47.6‰).

The δ^17^O measurements of ${\text{NO}}_{3}^{-}$ were conducted at the Stable Isotope Core Laboratory at Washington State University using the denitrifier method followed by thermal decomposition of nitrous oxide (N_2_O) (Kaiser et al., 2007; Komatsu et al., 2008) with analytical precision 0.84‰ for δ^18^O and 0.64‰ for δ^17^O (1σ, *n* =5) using the USGS34 and USGS35 (δ^17^O = 51.50‰ vs. VSMOW, δ^18^O = 56.81‰ vs. VSMOW) standards.

Isotopic data are reported with conventional delta notation vs. the international reference scales (air for N; VSMOW for O) in per mil (‰):


(1) $$\delta^{15}\text{N}={\left({{[}^{15}{\text{N}/}^{14}\text{N}]}_{\text{sample}}/{{[}^{15}{\text{N}/}^{14}\text{N}]}_{\text{air}}\;-\;1\right)}^{*}\;1000$$

(2) $$\delta^{18}\text{O}={\left({{[}^{\text{18}}{\text{O}/}^{16}\text{O}]}_{\text{sample}}/{{[}^{18}{\text{O}/}^{16}\text{O}]}_{\text{VSMOW}}\;-\;1\right)}^{*}\;1000$$

(3) $$\delta^{17}\text{O}={\left({{[}^{17}{\text{O}/}^{16}\text{O}]}_{\text{sample}}/{{[}^{17}{\text{O}/}^{16}\text{O}]}_{\text{VSMOW}}\;-\;1\right)}^{*}\;1000$$

### 2.4. Analysis of gaseous N species

The concentration of N_2_O was determined from gas sampled by a modified bubble strip method (protocol available at: http://dx.doi.org/10.17504/protocols.io.2x5gfq6). The N_2_O was measured with an HNU GC 301 gas chromatograph that was equipped with a Porapak N column under P-5 carrier gas (95% argon, 5% methane) at the USGS Water Mission Area Laboratories in Boulder as described in Repert et al. (2014) with a coefficient of variation for triplicate measurements of 11%.

### 2.5. Analysis of rock-N

Three peridotite rock core samples from the 280-meter depth interval in boreholes BA3A, BA4A, and BA1B of the multi-borehole observatory were obtained during Phase 2 of the Oman Drilling Project. Sampling procedures for clean retrieval of rock core are detailed in Templeton et al. (2021). Bulk δ^15^N of powdered peridotite was measured *via* continuous-flow isotope ratio mass spectrometry using the sealed tube combustion method (Boocock et al., 2020) on a Thermo Finnigan MAT253 in the St Andrews Stable Isotope Geochemistry (STAiG) laboratory. All three samples exhibited a signal/blank ratio >10:1.

### 2.6. N-cycling functional gene analysis

Metagenomic data for this study were previously published by Fones et al. (2019) and Kraus et al. (2021), including procedures regarding DNA extraction, metagenomic library prep, and sequencing. In short, DNA extraction was conducted according to the manufacturer's instructions with a MoBio PowerSoil Kit or Zymo Research Xpedition Soil/Fecal DNA MiniPrep extraction kit for samples collected in 2015 and 2017, respectively. Triplicate extractions were pooled, quantified, and normalized to 1 ng before library preparation using the Nextera XT kit. After tagmentation and amplification, products were pooled equimolarly and sequenced on an Illumina MiSeq platform (2x150 bp) at the University of Colorado Next-Generation Sequencing Facility (2015 samples) or an Illumina HiSeq 2,500 platform (2 x 250 bp) at the Duke Center for Genomic and Computational Biology (2017 samples).

Demultiplexed metagenomic sequences were merged (minimum length of 30), low-quality bases were trimmed off read ends (< 15), and reads of < 100 bases were discarded using the AdapterRemoval v2 (Schubert et al., 2016). Reads were quality filtered and then aligned to the NCycDB database (95% clustering) (Tu et al., 2019) for the identification of N-cycling genes using the Diamond aligner (Buchfink et al., 2015). Gene homolog abundances were normalized to metagenome size, and results from the two sampling years were combined.

### 2.7. Calculations of ^17^O difference (Δ^17^O)

The Δ^17^O can be determined by the following equation provided by Miller (2002):


(4) $$\begin{array}{l} \Delta^{17}O=\;\left[\ln\left(1+\;\frac{\delta^{17}O}{1000}\right)-0.52\cdot ln\left(1+\;\frac{\delta^{18}O}{1000}\right)\cdot 1000\;\right] \end{array}$$

The fraction of atmospheric endmember ${\text{NO}}_{3}^{-}$ (f_atm_) in an aquifer fluid can be calculated through a simple mass balance:


(5) $$\begin{array}{l} \Delta^{17}{\text{O}}_{\text{mixed}}=f_{\text{biogeo}}\left(\Delta^{17}{\text{O}}_{\text{biogeo}}\right)+f_{\text{atm}}\left(\Delta^{17}{\text{O}}_{\text{atm}}\right) \end{array}$$

where Δ^17^O_mixed_ is the ^17^O difference of ${\text{NO}}_{3}^{-}$ in a mixed aquifer fluid presumed to represent some fraction of atmospheric endmember ${\text{NO}}_{3}^{-}$ (Δ^17^O_atm_) and biogeochemical endmember ${\text{NO}}_{3}^{-}$ (Δ^17^O_biogeo_) ^17^O. The Δ^17^O_biogeo_ can be assumed to equal 0 because biogeochemical processes follow mass-dependent fractionation. Accordingly, equation (5) simplifies to:


(6) $$\begin{array}{l} f_{\text{atm}}=\Delta^{17}O_{\text{mixed}}/\left(\Delta^{17}O_{\text{atm}}\right) \end{array}$$

For Δ^17^O_atm_, we used the measured Δ^17^O of sampled rainwater. We recognize a single rainwater sample may not be entirely representative of the isotopic composition of mean annual rainfall since the Δ^17^O of dissolved NO_x_ in rainwater has been documented to fluctuate ~15‰ seasonally (Saud et al., 2022). The measured Δ^17^O is in the lower range expected for atmospheric deposition (Savard et al., 2018), with Δ^17^O values for atmospheric NO_x_ typically ranging between ~ 20–32‰ (Michalski et al., 2003). Accordingly, we applied an uncertainty of +15‰ for this endmember composition in mass balance calculations, using a Δ^17^O_atm_ of 31.8‰ to conservatively estimate f_atm_.

## 3. Results

### 3.1. Geochemical context of ophiolite fluids

Mg^2+^-${\text{HCO}}_{3}^{-}$ fluids were characterized by alkaline pH (8.3–9.2) and relatively high ∑Mg and ∑CO_2_ concentrations (0.37–3.3 mM and 1.3–3.6 mM, respectively) compared to the hyperalkaline pH (10–11.4) and high ∑Ca concentrations (0.43–7.8 mM) of Ca^2+^-OH^−^ fluids (Supplementary Table 1). The Ca^2+^-OH^−^ fluids also typically contained μM to mM concentrations of dissolved H_2_ and CH_4_. Of the wells sampled in this study, six wells hosted in peridotite were classified as Mg^2+^-${\text{HCO}}_{3}^{-}$ type fluids, representing open-system water–rock reaction under relatively oxidized conditions (Eh 78 to 180 mV), and seven wells as Ca^2+^-OH^−^ type fluids, representing closed-system water–rock reaction under highly reducing conditions (Eh as low as−415 mV).

**Figure 1:**
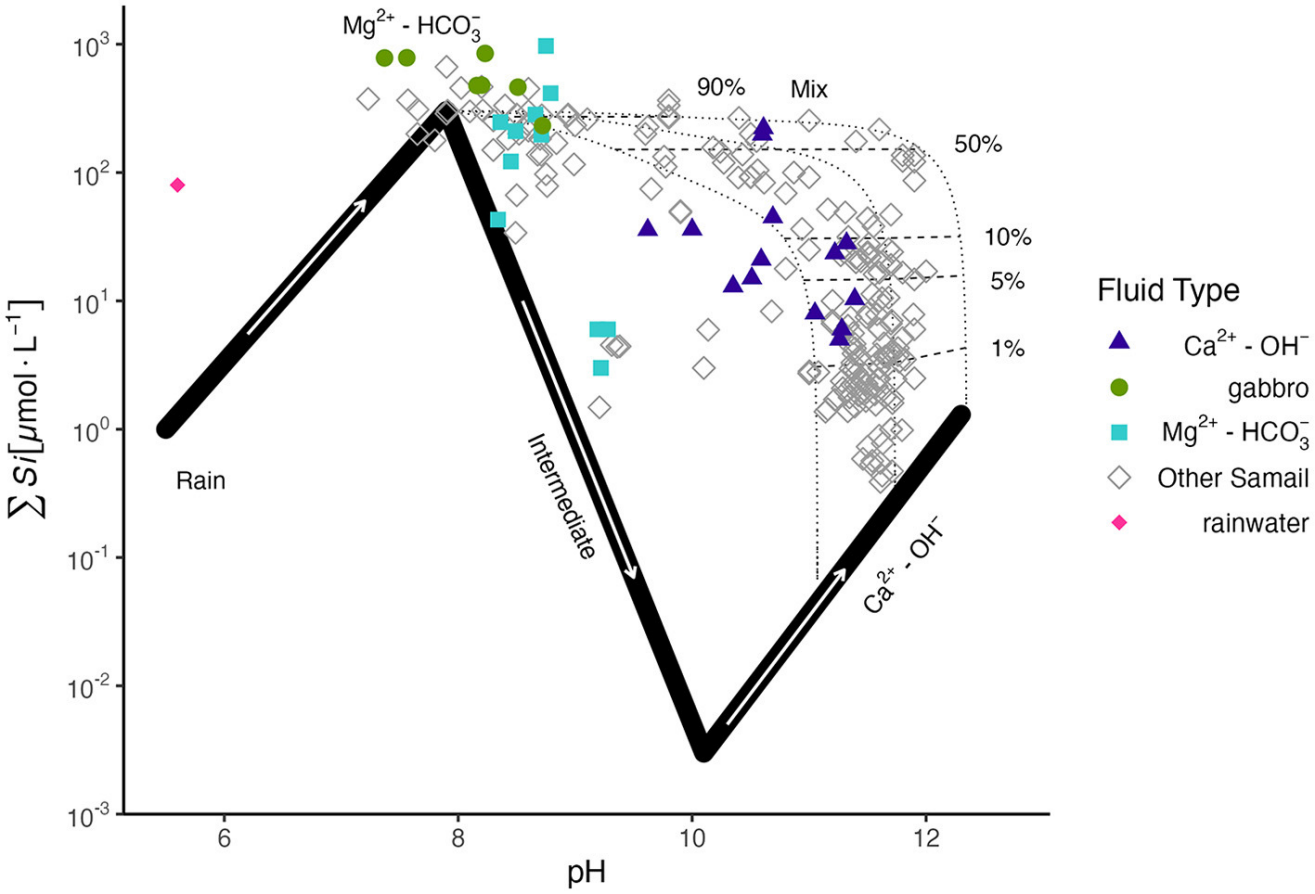
∑Si vs. pH of sampled fluids in this study, colored by fluid type, and of other Samail Ophiolite data (Stanger, 1986; Dewandel et al., 2004; Chavagnac et al., 2013; Rempfert et al., 2017; Leong et al., 2021) in gray, with the reaction path model of Leong et al. (2021) for the progressive reaction of rainwater with peridotite rock. Three potential Ca^2+^-OH^−^ compositions of varying ∑CO_2_ (8, 10, 20 μmole/kg from right to left) are plotted. ∑Si is used as a conservative tracer to distinguish the extent of mixing between Ca^2+^-OH^−^ and Mg^2+^-${\text{HCO}}_{3}^{-}$ fluids (shown in the plot as percentages next to mixing tie-lines). Mixing proportions are reported in Supplementary Table 2.

To assess the degree of mixing of deep, reacted Ca^2+^-OH^−^ fluids with less reacted Mg^2+^-${\text{HCO}}_{3}^{-}$ fluids in the near surface, we applied the Leong et al. (2021) approach of using ∑Si as a conservative tracer for the mixing of reacted fluids in ophiolitic aquifers. The composition of fluids collected in this study is plotted along the Leong et al. (2021) reaction path model (Figure 1) for the progressive reaction of rainwater with peridotite during serpentinization. No Ca^2+^-OH^−^ fluids sampled in this study displayed ∑Si concentrations as low as expected for chrysotile-brucite-calcite±diopside equilibrium, indicating some degree of mixing with Mg^2+^-${\text{HCO}}_{3}^{-}$ fluids. From our calculations of endmember fluid mixing (Supplementary Table 2), most Ca^2+^-OH^−^ fluids were mixed with < 10% Mg^2+^-${\text{HCO}}_{3}^{-}$ type fluids, except fluids sampled from BA1A from the 100–400 m packed interval and from well WAB56 which indicated mixing of ~15% and 57–73% Mg^2+^-${\text{HCO}}_{3}^{-}$ fluids, respectively. Fluids hosted within gabbro plotted with Mg^2+^-${\text{HCO}}_{3}^{-}$ fluids, but with slightly higher ∑Si concentrations.

### 3.2. Concentration and isotopic composition of dissolved N species

**Figure 2:**
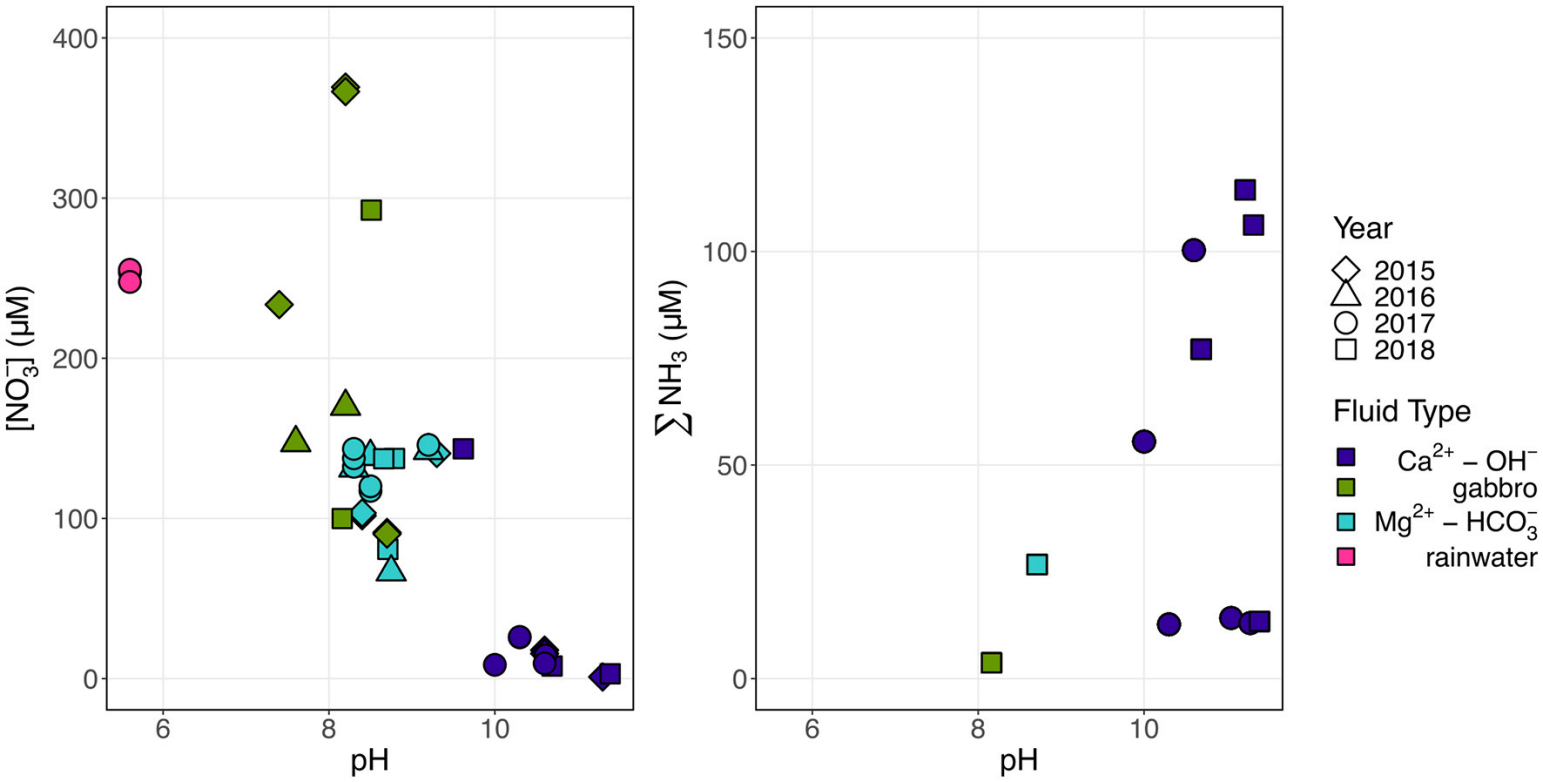
Trend of decreasing ${\text{NO}}_{3}^{-}$ concentrations and increasing ∑NH_3_ concentrations with pH. Colors indicate fluid composition and shapes indicate the year of sampling.

The predominant dissolved N species was dependent on the fluid type, with alkaline Mg^2+^-${\text{HCO}}_{3}^{-}$ type peridotite-hosted fluids and fluids hosted in gabbro comprised mostly of ${\text{NO}}_{3}^{-}$, and hyperalkaline Ca^2+^-OH^−^ peridotite-hosted fluids comprised primarily of reduced N (∑NH_3_) (Figure 2). The Mg^2+^-${\text{HCO}}_{3}^{-}$ type fluids contained ${\text{NO}}_{3}^{-}$ concentrations between 66 and 146 μM, while Ca^2+^-OH^−^ fluids only contained up to 26 μM (Table 2). Conversely, Ca^2+^-OH^−^ fluids were enriched in NH_3_ (up to 114 μM), while ∑NH_3_ concentrations were ~5 μM in all Mg^2+^-${\text{HCO}}_{3}^{-}$ type fluids (Table 3).

**Table 2:** δ^15^N and δ^18^O of ${\text{NO}}_{3}^{-}$ with ${\text{NO}}_{3}^{-}$ concentration measured by the denitrifier method.

**Sample**	**Year**	**Fluid type**	**${\text{NO}}_{3}^{-}$[μM]**	**δ^15^N ${\text{NO}}_{3}^{-}$‰**	**δ^18^O ${\text{NO}}_{3}^{-}$‰**
BA1A_100_400m	2018	Ca^2+^-OH^−^	7.8 ± 0.1	25.2 ± 0.09	34.6 ± 0.5
BA1A_55_66m	2018	Mg^2+^-${\text{HCO}}_{3}^{-}$	80.7 ± 3.2	6.1 ± 0.06	26.8 ± 0.3
NSHQ10	2016	Mg^2+^-${\text{HCO}}_{3}^{-}$	66.6 ± 1.1	20.1 ± 0.05	24.3 ± 0.3
NSHQ14	2015	Ca^2+^-OH^−^	1 ± 0.1	15.9 ± 0.09	20.6 ± 0.7
NSHQ14	2018	Ca^2+^-OH^−^	3 ± 0.1	5.6 ± 0.06	12.7 ± 0.5
NSHQ14_18m	2017	Ca^2+^-OH^−^	25.8 ± 1.4	10.6 ± 0.24	9.5 ± 0.3
NSHQ21	2015	Gabbro	233 ± 2.5	9.0 ± 0.07	14.9 ± 0.2
NSHQ3B	2015	Mg^2+^-${\text{HCO}}_{3}^{-}$	102.5 ± 1.1	2.8 ± 0.05	21.7 ± 0.2
NSHQ4	2017	Ca^2+^-OH^−^	8.6 ± 0.5	4.0 ± 0.2	19.5 ± 0.3
rainwater	2017	rainwater	252.2 ± 9.0	−2.2 ± 0.2	55.6 ± 0.3
WAB103	2015	Gabbro	367.9 ± 4	8.9 ± 0.06	16.7 ± 0.2
WAB103	2016	Gabbro	169.9 ± 2.1	11.8 ± 0.04	18.9 ± 0.2
WAB103	2018	Gabbro	292.5 ± 4.9	10.5 ± 0.06	18.1 ± 0.3
WAB104	2016	Mg^2+^-${\text{HCO}}_{3}^{-}$	138.6 ± 2	1.4 ± 0.03	22.9 ± 0.2
WAB104	2017	Mg^2+^-${\text{HCO}}_{3}^{-}$	118.6 ± 5.9	2.3 ± 0.2	22.9 ± 0.3
WAB104	2018	Mg^2+^-${\text{HCO}}_{3}^{-}$	137.5 ± 3.4	1.1 ± 0.06	23.5 ± 0.3
WAB105	2016	Mg^2+^-${\text{HCO}}_{3}^{-}$	131.4 ± 1.5	2 ± 0.03	23.6 ± 0.2
WAB105	2017	Mg^2+^-${\text{HCO}}_{3}^{-}$	137.8 ± 4.9	2.5 ± 0.1	21.8 ± 0.6
WAB105	2018	Mg^2+^-${\text{HCO}}_{3}^{-}$	137.3 ± 3.1	2.2 ± 0.06	22 ± 0.3
WAB188	2015	Gabbro	90.7 ± 1	5.5 ± 0.05	22.5 ± 0.2
WAB188	2016	Gabbro	147.3 ± 1.7	3.3 ± 0.03	21.8 ± 0.2
WAB188	2017	Gabbro	135.9± 6.2	5.5 ± 0.04	22.5 ± 0.2
WAB188	2018	Gabbro	99.9 ± 3.1	3.2 ± 0.06	21.3 ± 0.3
WAB55	2015	Mg^2+^-${\text{HCO}}_{3}^{-}$	140 ± 1.5	7.4 ± 0.07	21.1 ± 0.2
WAB55	2016	Mg^2+^-${\text{HCO}}_{3}^{-}$	142.3 ± 1.7	7.5 ± 0.03	20.8 ± 0.2
WAB55	2017	Mg^2+^-${\text{HCO}}_{3}^{-}$	145.9 ± 0.9	8.8 ± 0.05	20.9 ± 0.2
WAB55	2018	Ca^2+^-OH^−^	143.5 ± 2.9	7.8 ± 0.06	20.5 ± 0.3
WAB56	2015	Ca^2+^-OH^−^	16.7 ± 0.3	20.7 ± 0.09	30.7 ± 0.2
WAB56	2017	Ca^2+^-OH^−^	14.4 ± 0.8	9.94 ± 0.23	22.8 ± 0.3
WAB71	2017	Ca^2+^-OH^−^	9.5 ± 0.5	13.2 ± 0.26	0.9 ± 0.3

Standard error is calculated from all experimental replicates.

**Table 3:** δ^15^N of N_red_ which represents the total reduced nitrogen in the sampled fluid (measured by the denitrifier method through mass balance after persulfate oxidation).

**Sample**	**Fluid type**	**Year sampled**	**N_red_ [μM]**	**∑NH_3_ [μM]**	**δ^15^N_red_ ‰**
BA1A_100_400m	Ca^2+^-OH^−^	2018	80.2 ± 7.6	77.1 ± 10.0	−12.9 ± 1.5
BA1A_55_66m	Mg^2+^-${\text{HCO}}_{3}^{-}$	2018	32.0 ± 12.3	26.7 ± 0.5	−16.7 ± 6.5
CM2A	Ca^2+^-OH^−^	2018	99.1 ± 8.8	106.2 ± 5.9	6.9 ± 0.9
NSHQ14	Ca^2+^-OH^−^	2018	28.3 ± 2.4	13.4 ± 2.4	4.6 ± 0.5
NSHQ14_18m	Ca^2+^-OH^−^	2017	20.4 ± 1.2	12.7 ± 0.4	5.0 ± 0.6
NSHQ14_50m	Ca^2+^-OH^−^	2017	23.0 ± 0.4	14.2 ± 0.3	9.6 ± 0.2
NSHQ14_85m	Ca^2+^-OH^−^	2017	18.1 ± 0.3	13.0 ± 0.3	4.3 ± 0.2
NSHQ4	Ca^2+^-OH^−^	2017	50.7 ± 1.2	55.5 ± 5.0	0.9 ± 0.1
WAB188	Gabbro	2018	41.8 ± 14.5	3.7 ± 1.0	2.4 ± 1.4
WAB56	Ca^2+^-OH^−^	2017	141.6 ± 2.4	*-*	3.4 ± 0.1
WAB71	Ca^2+^-OH^−^	2017	109.6 ± 2.0	100.3 ± 1.9	11.2 ± 0.3
WAB71	Ca^2+^-OH^−^	2018	105.6 ± 11.1	114.4 ± 3.1	13.6 ± 2.0

Standard error is calculated from all experimental replicates. Concentrations of NH_3_ via colorimetric assay are provided for comparison.

Fluids hosted within gabbros were also dominated by ${\text{NO}}_{3}^{-}$, but with higher concentrations (as high as 366 μM) than observed for Mg^2+^-${\text{HCO}}_{3}^{-}$ fluids. Both Mg^2+^-${\text{HCO}}_{3}^{-}$ and Ca^2+^-OH^−^ type fluids had lower concentrations of ${\text{NO}}_{3}^{-}$ than observed in rainwater (252 μM); however, gabbro well WAB103 demonstrated concentrations of ${\text{NO}}_{3}^{-}$ greater than rainwater. ${\text{NO}}_{2}^{-}$ was often detectable across fluid types, but in very low concentrations (~1 μM) except for in a few fluids where ${\text{NO}}_{2}^{-}$ was present at concentrations between ~4 and 30 μM (Table 4). All wells where dissolved N_2_O concentrations were analyzed contained detectable N_2_O, which varied in concentration from 5 to 177 nM, with the highest concentration observed in the packed-off interval 55–66 m in borehole BA1A (Table 5).

**Table 4:** δ15N and δ18O of ${\text{NO}}_{2}^{-}$ with ${\text{NO}}_{2}^{-}$ concentration measured by mass balance by the denitrifier method after ${\text{NO}}_{2}^{-}$ removal with sulfamic acid.

**Sample**	**Year**	**Fluid type**	**${\text{NO}}_{2}^{-}$[μM]**	**δ^15^N ${\text{NO}}_{2}^{-}$‰**	**δ^18^O ${\text{NO}}_{2}^{-}$‰**
NSHQ10	2016	Gabbro	30.3 ± 1.6	−17.9 ± 1.3	5.2 ± 1.7
NSHQ4	2017	Ca^2+^-OH^−^	4.8 ± 0.8	−4.4 ± 1.0	21.1 ± 3.1
WAB56	2015	Ca^2+^-OH^−^	3.8 ± 0.4	7.8 ± 2.3	22.5 ± 3.3

Errors reported are propagated errors for replicate analyses.

**Table 5:** Concentrations of dissolved N_2_O measured in gases collected from the 2018 fluids *via* the bubble strip method.

**Sample**	**Fluid type**	**N_2_O_(g)_ [nM]**
BA1A-100-400m	Ca^2+^-OH^−^	5.05E00
BA1A-55-66m	Mg^2+^-${\text{HCO}}_{3}^{-}$	1.77E02
CM2A	Ca^2+^-OH^−^	5.13E00
NSHQ14	Ca^2+^-OH^−^	8.70E00
WAB103	gabbro	2.45E01
WAB104	Mg^2+^-${\text{HCO}}_{3}^{-}$	2.03E01
WAB105	Mg^2+^-${\text{HCO}}_{3}^{-}$	1.67E01
WAB188	gabbro	1.26E01
WAB55	Ca^2+^-OH^−^	1.87E01
WAB71	Ca^2+^-OH^−^	1.47E01

**Figure 3:**
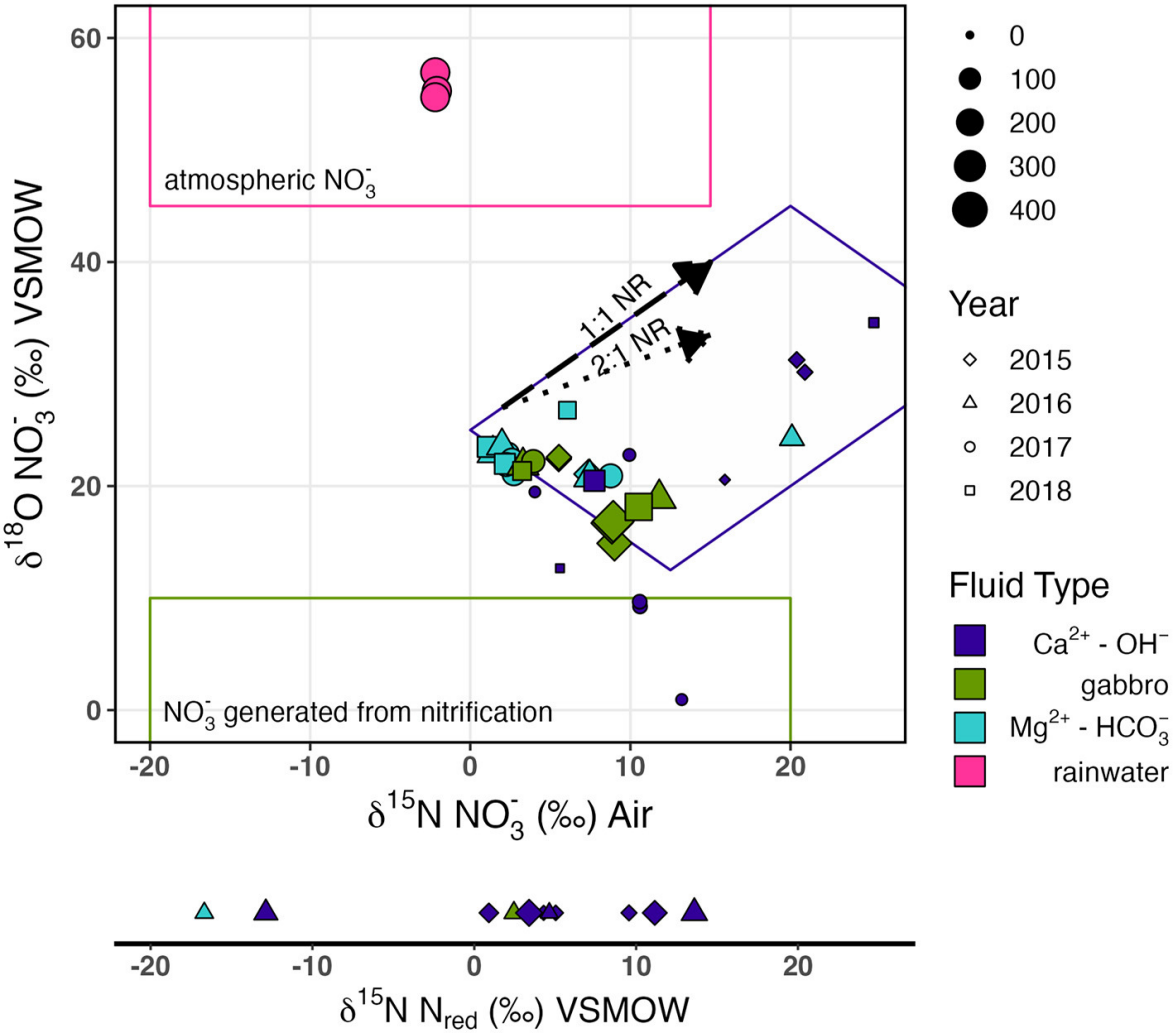
δ^18^O vs. δ^15^N of ${\text{NO}}_{3}^{-}$ biplot, with the δ^15^N of reduced N plotted on the same scale. Colors indicate fluid type, shapes indicate the year of sampling, and the size of points represents the concentration of N species in μM. Trends for NR with a slope of 1 (labeled 1:1; long dash) and 0.5 (labeled 2:1 with dotted arrow) are illustrated. The field outlined in pink represents the common isotopic composition for atmospheric ${\text{NO}}_{3}^{-}$, the field outlined in green represents the common isotopic composition of nitrified ${\text{NO}}_{3}^{-}$, and the field outlined in purple represents samples with ^15^N- and ^18^O-enriched isotopic compositions consistent with NR. Isotopic fields for common isotopic compositions were sourced from Kendall et al. (2007).

The dual isotopic composition of ${\text{NO}}_{3}^{-}$ (δ^15^N and δ^18^O) offers valuable information on potential sources and subsequent transformations of ${\text{NO}}_{3}^{-}$ in the subsurface, aquifer ecosystem. On a biplot of δ^15^N and δ^18^O of ${\text{NO}}_{3}^{-}$ (Figure 3), Oman rainwater plots within the range expected for atmospheric deposition (Oman rainwater: δ^15^N −2.2‰, δ^18^O 55.6‰) (Kendall Carol, 1998; Kendall et al., 2007). A few Ca^2+^-OH^−^ fluids, where ${\text{NO}}_{3}^{-}$ concentrations are < 26 μM, plot within the range expected for nitrification-derived ${\text{NO}}_{3}^{-}$ (δ^18^O < 10‰) (Kendall Carol, 1998; Kendall et al., 2007). However, most Samail ophiolite aquifer fluids exhibit δ^18^O between these two sources, indicating the likely contribution of ${\text{NO}}_{3}^{-}$ from both atmospheric and biological nitrification sources.

**Figure 4:**
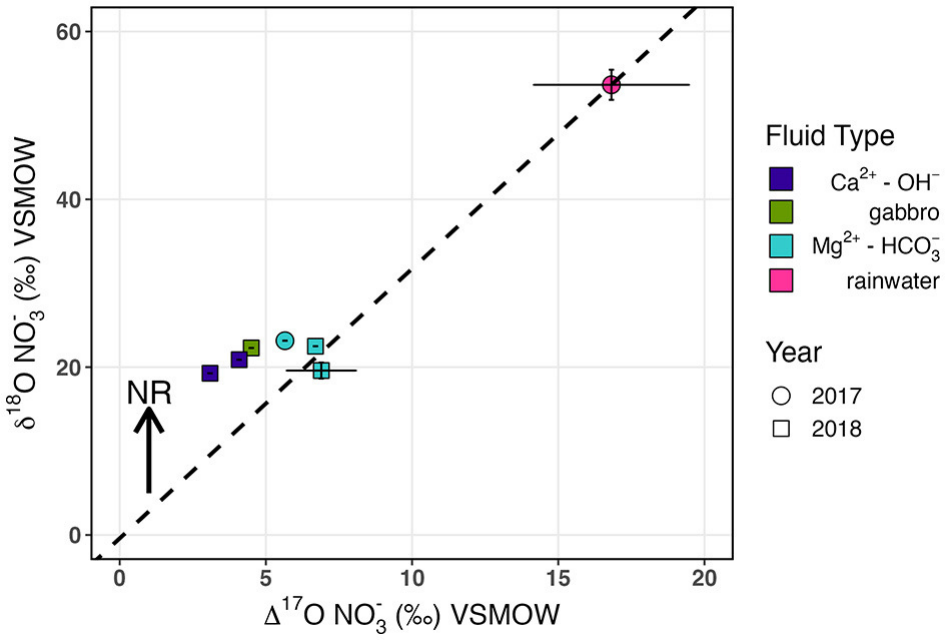
δ^18^O vs. Δ^17^O of ${\text{NO}}_{3}^{-}$. Mg^2+^-${\text{HCO}}_{3}^{-}$ fluids (blue) plot on a theoretical mixing line with rainwater (pink) calculated for simple mixing of estimated end-member nitrified ${\text{NO}}_{3}^{-}$ (δ^18^O of−0.4‰ and Δ^17^O of 0‰). ${\text{NO}}_{3}^{-}$ reduction would lead to increased δ^18^O with no change in Δ^17^O.

To investigate atmospheric deposition as a potential source of ${\text{NO}}_{3}^{-}$ to the aquifer, we measured the δ^17^O of oxidized aqueous N species (NO_x_ = ${\text{NO}}_{2}^{-}$ + ${\text{NO}}_{3}^{-}$) in a subset of samples from the 2017 and 2018 field seasons. Atmospherically sourced ${\text{NO}}_{3}^{-}$ and ${\text{NO}}_{2}^{-}$ have δ^17^O higher than predicted for mass-dependent fractionation due to photochemical reactions with ozone in the stratosphere (Thiemens, 1999, 2006; Lyons, 2001; Mauersberger et al., 2001; Michalski et al., 2002, 2003, 2004). Because subsequent biological fractionation of atmospherically derived ${\text{NO}}_{3}^{-}$ should not impact the deviation of δ^17^O in oxidized N species from expected mass-dependent fractionation (Δ^17^O of NO_x_), the Δ^17^O of measured NO_x_ can be used to calculate the relative contribution of biogeochemical and atmospheric sources of ${\text{NO}}_{3}^{-}$ (Michalski et al., 2003; Riha et al., 2014). Biogeochemical sources, such as nitrification-derived ${\text{NO}}_{3}^{-}$, are assumed to have a mass-dependent Δ^17^O value of 0‰. We found that all measured fluids contained ${\text{NO}}_{3}^{-}$ which reflected some contribution of a relict atmospheric source, with Δ^17^O above 0‰ (Table 6). The Δ^17^O was highest in Mg^2+^-${\text{HCO}}_{3}^{-}$ type peridotite-hosted fluids (Figure 4) and generally correlated with the δ^18^O and concentration of ${\text{NO}}_{3}^{-}$. These Δ^17^O corresponded to estimated fractions of atmospheric endmember ${\text{NO}}_{3}^{-}$ (f_atm_) ranging from 0.09 to 0.41, accounting for uncertainty in the endmember Δ^17^O_atm_ isotopic composition.

**Table 6:** δ^17^O and δ^18^O isotopic compositions of ${\text{NO}}_{3}^{-}$.

**Sample**	**Year sampled**	**δ^18^O ${\text{NO}}_{3}^{-}$(‰)**	**δ^17^O ${\text{NO}}_{3}^{-}$(‰)**	**Δ^17^O ${\text{NO}}_{3}^{-}$(‰)**	**f_atm_**
Rainwater	2017	53.7 (1.8)	44.7 (3.6)	16.8 (2.7)	1 [0.47]
WAB104	2018	19.6 (1.0)	17.1 (0.7)	6.9 (1.2)	0.41 [0.19]
BA1A 100-400m packed interval	2018	32.8	21.3	4.3	0.25 [0.12]
BA1A 55-66m packed interval	2018	26.4	20.7	6.9	0.41[0.19]
NSHQ14	2018	19.3	13.1	3.1	0.18 [0.09]
WAB104	2017	23.2	17.7	5.7	0.34 [0.16]
WAB105	2018	22.5	18.4	6.7	0.40 [0.19]
WAB188	2018	22.3	16.1	4.5	0.27 [0.13]
WAB55	2018	20.89	15.0	4.09	0.24 [0.11]

^17^O difference (Δ^17^O) and corresponding calculated atmospheric endmember fractional contribution to the observed Δ^17^O signatures of ${\text{NO}}_{3}^{-}$ in borehole fluids are also reported. Errors (1σ, n = 2) for replicate measures are displayed in parentheses. Conservative estimates of f_atm_, displayed in brackets, were calculated by applying an uncertainty of 15‰ for expected endmember Δ^17^O_atm_ composition.

By mass balance, the isotopic composition of biogeochemically derived ${\text{NO}}_{3}^{-}$, δ^18^O ${\text{NO}}_{3}^{-}$_biogeo_, can be calculated (Supplementary Table 3). The δ^18^O ${\text{NO}}_{3}^{-}$_biogeo_ reflects ${\text{NO}}_{3}^{-}$ produced by nitrification as well as processes that act to enrich the ${\text{NO}}_{3}^{-}$ pool, such as nitrate reduction (NR) during biological assimilation or respiration. Most measured samples exhibited δ^18^O ${\text{NO}}_{3}^{-}$_biogeo_ < 10‰, consistent with nitrification-derived ${\text{NO}}_{3}^{-}$ (Kendall et al., 2007; Xue et al., 2009; Kaushal et al., 2011; Yi et al., 2017). However, fluids collected from borehole BA1A with the packer system to isolate deep Ca^2+^-OH fluids below 100 m had δ^18^O of 27‰, suggesting extensive NR. Biological NR causes the δ^18^O and δ^15^N of ${\text{NO}}_{3}^{-}$ in the residual pool to increase in a relatively predictive pattern, with the proportionality of N and O isotopic fractionation varying between 0.5 and 1 (Böttcher et al., 1990; Sigman et al., 2005; Granger et al., 2008; Chen and MacQuarrie, 2011; Knöller et al., 2011; Granger and Wankel, 2016; Asamoto et al., 2021). We observed a general trend of coupled increase in δ^18^O and δ^15^N of ${\text{NO}}_{3}^{-}$ with a proportionality of ~1 (Figure 3), consistent with NR.

The δ^15^N of reduced nitrogen species (N_red_) spanned a range of 30‰ (Table 4). In most samples, ∑NH_3_ concentrations were comparable to the concentration of N_red_ measured *via* the mass balance of total N after persulfate oxidation compared to total oxidized nitrogen species (NO_*x*_ = ${\text{NO}}_{3}^{-}$ + ${\text{NO}}_{2}^{-}$); however, this was not true in samples from WAB188 and NSHQ14 where concentrations of N_red_ were greater than measured ∑NH_3_. In these wells, the δ^15^N of N_red_ must be interpreted as a mixture of ∑NH_3_ and other dissolved forms of reduced N, such as organic compounds. The δ^15^N of N_red_ varied considerably, with the highest δ^15^N (13.6‰) observed for borehole WAB71 where the concentration of NH_3_ was greatest (114 μM).

### 3.3. Bulk rock N abundance and δ^15^N

Nitrogen can be stored in rocks as recalcitrant organic matter, ${\text{NH}}_{4}^{+}$ or ${\text{NO}}_{3}^{-}$ salts, nitride minerals, substituted in hydrous minerals, incorporated into the structure of silicate minerals, or as gas within fluid inclusions (Holloway and Dahlgren, 2002; Loganathan and Kalinichev, 2013; Mysen, 2019). Because this N could be liberated or assimilated during water–rock reaction (Silver et al., 2012; Houlton et al., 2018), we measured the abundance of bulk N in peridotite rock using the sealed tube combustion method which allows for the measurement of even stably bound N within the silicate mineral structure (Bebout et al., 2007; Boocock et al., 2020). N abundances were low (11.3–13.9 ppm) in analyzed peridotite rock core samples and bulk δ^15^N of peridotite samples varied from 3.5 to 6.7‰ (Table 7).

**Table 7:** Bulk rock δ15N of peridotites sampled at the 280-m depth interval in boreholes BA1B, BA3A, and BA4A.

**Borehole**	**Depth [m]**	**ppm N**	**δ^15^N N_bulk_ ‰**
BA1B	280	11.3	4.4
BA3A	280	13.9	3.54
BA4A	280	11.3	6.66

### 3.4. Presence of N-cycling genes

**Figure 5:**
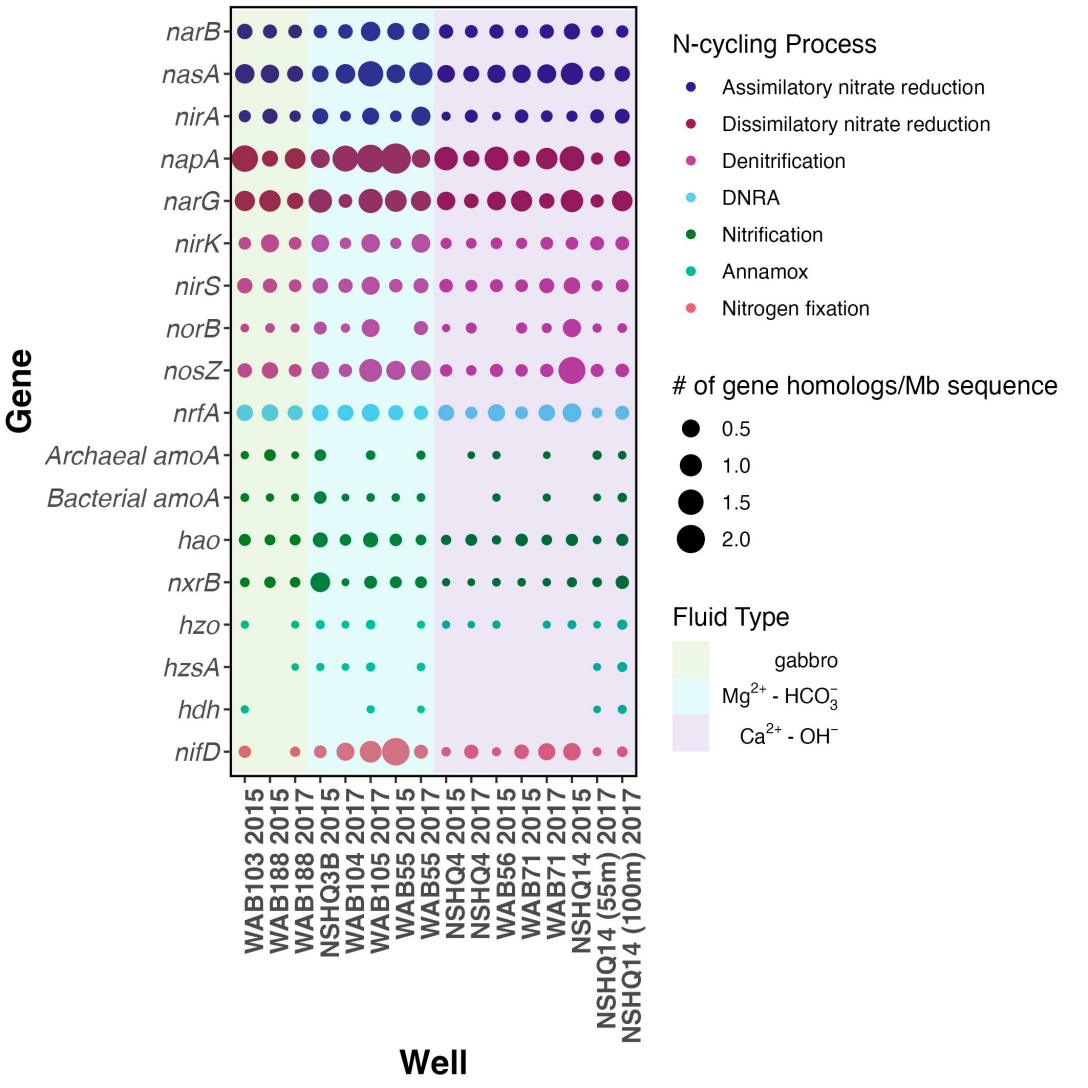
Dotplot of abundances of key N cycling genes, where the colors of the dots correspond to the type of N-cycling process, the size to the # of gene homologs detected per Mb sequence, and the background color representative of fluid type.

To assess the possibility of microbial involvement in the cycling of N within Samail Ophiolite aquifers, we used metagenomic sequencing of groundwater fluid biomass to probe for genes known to be involved in N utilization or transformation (Figure 5). N-cycling gene homologs were fairly ubiquitous across sampled fluids, albeit in low abundance (< 2 gene homologs/Mb of sequence). Gene homologs associated with NR, both assimilatory *narB* and dissimilatory reductases *narG* and *napA*, were most abundant although homologs for the cytochrome c nitrite reductase (*nrfA*) involved in the reduction of ${\text{NO}}_{2}^{-}$ to ${\text{NH}}_{4}^{+}$ in dissimilatory nitrate reduction to ammonium (DNRA) were also notably abundant across fluids. We did not observe any major trends in gene absence or presence by fluid type.

It is important to note that these methods only detect the presence of these genes in aquifer fluids, and do not indicate whether individual organisms possess all genes involved in any specific N-cycling pathway, or whether these genes are actively expressed or utilized. Nevertheless, the presence of gene homologs for N-cycling processes in Samail Ophiolite fluids suggests the potential for biological transformations.

## 4. Discussion

### 4.1. Rainwater delivers atmospheric N to Samail Ophiolite aquifers

The positive measured Δ^17^O of ${\text{NO}}_{3}^{-}$ for all sampled groundwaters suggests atmospheric deposition is a primary source of N to Samail Ophiolite aquifers. The degree to which ${\text{NO}}_{3}^{-}$ is sourced from the atmosphere can be estimated using the δ^17^O of ${\text{NO}}_{3}^{-}$ as a conservative tracer. During ozone formation, the ratio of ^18^O/^16^O becomes equally elevated as the ratio of ^17^O/^16^O, thus enriching both isotopes independent of their mass difference (Thiemens and Heidenreich, 1983; Thiemens et al., 2001; Miller, 2002). Ozone then transfers O atoms during oxidation reactions that result in a positive Δ^17^O of many oxygen sources in the atmosphere (O_3_, O_2_, H_2_O) which can be inherited by N-oxides during photochemical reaction (typically Δ^17^O of 20–32‰) (Savarino et al., 2000, 2008; Lyons, 2001; Michalski et al., 2012). Mixing with biogeochemically sourced ${\text{NO}}_{3}^{-}$ would lower Δ^17^O toward 0‰ (Casciotti et al., 2002; Michalski et al., 2003; Ewing et al., 2007; Kendall et al., 2007; Dejwakh et al., 2012; Riha et al., 2014). We measured a range of Δ^17^O for groundwater ${\text{NO}}_{3}^{-}$ of 3.1 to 6.9‰, corresponding to estimated atmospheric endmember contributions of 18 to 41% using a simple two-member isotope mixing model (Table 6).

This atmospheric contribution represents both wet and dry deposition. The concentration of ${\text{NO}}_{3}^{-}$ in rainwater was high (~255 μM), but precipitation in Oman is scarce and sporadic, with elevations below 1000 m typically receiving only 60–100 mm of rainfall per year from mostly Mediterranean frontal systems (Weyhenmeyer et al., 2002). Accordingly, dry deposition may contribute a more significant flux of N to Samail Ophiolite aquifers compared to wet deposition. While not well constrained, up to 82% of atmospheric NO_x_ deposition occurs as dry deposition in arid regions of central Asia (Li et al., 2013). An atmospheric origin of ${\text{NO}}_{3}^{-}$ is consistent with reports for soil crusts in desert environments such as the Mojave and the Atacama as well as in catchments in the southwestern United States where 31 to 100% of soil nitrate and up to 82% of stream nitrate is atmospherically derived (Böhlke et al., 1997; Michalski et al., 2004; Lybrand et al., 2013; Riha et al., 2014).

A partial atmospheric source for the measured ${\text{NO}}_{3}^{-}$ in shallow aquifer fluids would require effective transference of N to the subsurface. Despite low annual rainfall, the effective rainfall in the Samail Ophiolite is relatively high. In a hydrologic study conducted in the Ibra region of the Samail Ophiolite by Dewandel et al. (2005), 50 mm of rain per year was estimated to be effective rain, of which 18 ± 8 mm is presumed to recharge shallow peridotite aquifers. Estimated recharge into gabbro is predicted to be even higher (>20 mm/year) on account of the greater hydraulic conductivity of gabbros compared to peridotites in the Samail Ophiolite (10^−5^ to 10^−6^ for gabbro and 10^−7^ for serpentinized peridotite) and the likely additional input of surface runoff due to the moderate relief of gabbro outcrops, both of which could explain the higher ${\text{NO}}_{3}^{-}$ concentrations observed in some gabbro wells (Dewandel et al., 2005). The absence of soil or major vegetation in this environment further facilitates the rapid transfer of rainwater to the subsurface before significant biological processing can remove N from the infiltrating fluids.

### 4.2. Nitrification-derived ${\text{NO}}_{3}^{-}$ comprises the rest of the groundwater ${\text{NO}}_{3}^{-}$ Pool

The remaining 59–91% of subsurface ${\text{NO}}_{3}^{-}$ can be explained by biological nitrification. Nitrification is one of the two biological processes that produce ${\text{NO}}_{3}^{-}$ during the biogeochemical cycling of N (Granger and Wankel, 2016). In this two-step process, NH_3_ is oxidized to ${\text{NO}}_{2}^{-}$ and then ${\text{NO}}_{3}^{-}$, coupled with aerobic respiration (Verstraete and Focht, 1977; Teske et al., 1994). Both bacteria and archaea mediate NH_3_ oxidation, but only bacteria are known to carry out the ${\text{NO}}_{2}^{-}$ oxidation step, with some bacterial taxa capable of completely oxidizing NH_3_ to ${\text{NO}}_{3}^{-}$ (comammox) (Daims et al., 2015; van Kessel et al., 2015). We detected both archaeal and bacterial functional markers for NH_3_ oxidation (*amoA* gene), with no differential abundance by fluid type, despite ∑NH_3_ concentration acting as a strong selector for NH_3_ oxidizing taxa in other environments (Martens-Habbena et al., 2009; Bates et al., 2011; Verhamme et al., 2011; Lehtovirta-Morley, 2018). NH_3_ oxidation is presumed to be primarily aerobic because we only sparsely detected gene homologs for hydrazine dehydrogenase (*hdh*), which encodes the key enzyme in anaerobic ammonia oxidation (anammox) for catalyzing hydrazine oxidation to N_2_ gas (Kartal et al., 2007; Maalcke et al., 2016) (see Supplementary datasheet).

In NH_3_ oxidation to ${\text{NO}}_{2}^{-}$, O is incorporated enzymatically from O_2_ and H_2_O in a 1:1 ratio, whereas in ${\text{NO}}_{2}^{-}$ oxidation to ${\text{NO}}_{3}^{-}$, O is solely derived from H_2_O (Andersson and Hooper, 1983; Buchwald and Casciotti, 2010). The δ^18^O of nitrified ${\text{NO}}_{3}^{-}$ has been shown to closely resemble the isotopic composition of ambient H_2_O in both labeled nitrifying incubations (Boshers et al., 2019) and field observations (Buchwald and Casciotti, 2010; Buchwald et al., 2012). Accordingly, we presume that nitrified ${\text{NO}}_{3}^{-}$ in Samail Ophiolite aquifers should have a δ^18^O of−0.4‰, which represents the average δ^18^O of aquifer H_2_O measured from a subset of 2018 Mg^2+^-${\text{HCO}}_{3}^{-}$ and Ca^2+^-OH^−^ fluids (Supplementary Table 4). Utilizing this estimated value of−0.4‰ for δ^18^O of ${\text{NO}}_{3}^{-}$ along with a Δ^17^O of 0‰ as a nitrification “end-member” for Samail Ophiolite fluids, we applied a simple two-member isotope mixing model with ${\text{NO}}_{3}^{-}$ in rainwater (${\text{NO}}_{3}^{-}$ δ^18^O 53.7‰, Δ^17^O 16.8‰) (Figure 4). We can replicate the observed δ^18^O and Δ^17^O of shallow aquifer ${\text{NO}}_{3}^{-}$ by solely mixing end-member atmospheric and nitrification sources, as all Mg^2+^-${\text{HCO}}_{3}^{-}$ fluids plotted close to the predicted mixing line. Ca^2+^-OH^−^ fluids plotted closer to the estimated nitrification source, which likely represents Ca^2+^-OH^−^ fluids containing a greater proportion of nitrified ${\text{NO}}_{3}^{-}$ than Mg^2+^-${\text{HCO}}_{3}^{-}$ fluids.

### 4.3. ${\text{NO}}_{3}^{-}$ is extensively reduced to NH_3_ during progressive water–rock reaction

The concomitant decrease in ${\text{NO}}_{3}^{-}$ concentration and increase in ∑NH_3_ concentration with increasing pH (Figure 2) suggests a possible reduction of ${\text{NO}}_{3}^{-}$ to ∑NH_3_ during the progressive reaction of Mg^2+^-${\text{HCO}}_{3}^{-}$ type fluids to reducing, Ca^2+^-OH^−^ type fluids. We observed a trend of increasing δ^15^N and δ^18^O of ${\text{NO}}_{3}^{-}$ with decreasing ${\text{NO}}_{3}^{-}$ concentration (Figure 3) indicative of biological NR which characteristically enriches δ^15^N and δ^18^O of residual nitrate in approximately a 1:1 to 2:1 ratio (Casciotti and McIlvin, 2007; Casciotti et al., 2013; Gaye et al., 2013; Rafter et al., 2013; Bourbonnais et al., 2017). This trend implies ^15^N isotopic discrimination of NR between −4.5‰ and −17.5‰ (Supplementary Figure 1) assuming Rayleigh kinetic fractionation dynamics. It is important to note that Samail Ophiolite aquifer fluids do not satisfy all assumptions for the Rayleigh fractionation model because nitrification and diffusion of ${\text{NO}}_{3}^{-}$ from near-surface mixing may continually supply some ${\text{NO}}_{3}^{-}$ during ${\text{NO}}_{3}^{-}$ consumption.

In the Samail Ophiolite, reductants such as H_2_ are produced during water-rock interactions, and most oxidants are highly limited in reacted hyperalkaline fluids. Thus, we would predict that ${\text{NO}}_{3}^{-}$ introduced into the aquifer would act as an important electron acceptor for subsurface microbial metabolism. Accordingly, microbial NR through DNRA is a likely explanation for the presumed production of ∑NH_3_ in Samail Ophiolite aquifer fluids. DNRA is a common respiratory process in oligotrophic marine (Lam et al., 2009; Bonaglia et al., 2016), soil (Silver et al., 2001; Rütting et al., 2011; Zhang et al., 2015), and freshwater riparian to estuarine wetland environments (Welsh et al., 2001; Koop-Jakobsen and Giblin, 2010; Wang et al., 2020). The ubiquitous detection of *nrfA* gene homologs, the gene encoding the catalytic subunit for cytochrome c ${\text{NO}}_{2}^{-}$ reductase for ${\text{NO}}_{2}^{-}$ reduction to ${\text{NH}}_{4}^{+}$, further supports a biological role in the production of subsurface ∑NH_3._

While the isotope effect for DNRA has not been systematically evaluated, it is predicted to be similar to that of denitrification with an estimated maximum fractionation of ~−30‰ (McCready et al., 2011). In both denitrification and DNRA, ${\text{NO}}_{3}^{-}$ is first reduced by the periplasmic enzyme Nap (catalytic subunit encoded by *napA*) or the membrane-bound cytosolic enzyme Nar (catalytic subunit encoded by *narG*) which have ^15^ε ranges of −11.4 to −39.8‰ and −6.6 to −31.6‰, respectively (Granger and Wankel, 2016; Asamoto et al., 2021; and references therein). There is an enzyme-specific coupling of O and N isotope fractionation during NR, with Nar reductases commonly imparting fractionation with a ^18^ε/^15^ε proportionality of approximately 0.91, and Nap reductases a ^18^ε/^15^ε proportionality of ~ 0.55 (Asamoto et al., 2021). Accordingly, we presume if NR in Samail Ophiolite aquifer fluids is microbial, Nar reductases best explain the demonstrated proportionality of enrichment in δ^18^O vs. δ^15^N which was close to 1 (Figure 3). The observed discrimination for NR (−4.5 to −17.5‰) in Samail Ophiolite aquifers is largely consistent with dissimilatory NR with a Nar reductase, with reduced isotope effects due to ${\text{NO}}_{3}^{-}$ uptake becoming the rate-limiting step at low concentrations of ${\text{NO}}_{3}^{-}$ (Kritee et al., 2012) (see Supplementary datasheet). The predominance of Nap reductases in addition to Nar reductases in Samail Ophiolite aquifers could be explained by the functional diversity of Nap enzymes which can additionally be involved in the maintenance of cellular oxidation-reduction potential and ${\text{NO}}_{3}^{-}$ scavenging (Potter and Cole, 1999; Potter et al., 1999; Richardson, 2000). Accordingly, the abundance of *napA* gene homologs does not necessarily indicate an active role in dissimilatory NR.

We do also consider that an abiotic reduction process could play a role in the conversion of ${\text{NO}}_{3}^{-}$ to ∑NH_3_. Although spontaneous NR in the presence of high H_2_ concentrations is not expected to occur at the temperatures of the aquifer fluids (~35° C), mineral-facilitated reduction by Fe-bearing phases could occur. For example, the quantitative conversion of ${\text{NO}}_{3}^{-}$ to ${\text{NH}}_{4}^{+}$ can be catalyzed by green rust minerals at surface temperatures (Hansen et al., 1996). Similarly, Smirnov et al. (2008) reported the generation of ${\text{NH}}_{4}^{+}$ through NR in the presence of the FeNi alloys, such as awaruite (Ni_80_Fe_20_), although this reaction was highly temperature-dependent and proceeded almost negligibly at 22 °C (Smirnov et al., 2008). Yet, the detection of awaruite and Fe-bearing hydroxides in serpentinized peridotite in the Samail Ophiolite (Ellison et al., 2021) merits future investigation into the kinetics and associated isotope effects of these reactions under environmentally relevant conditions.

We note that other biological reduction pathways including denitrification to N_2_ or assimilatory NR may have contributed to the observed ${\text{NO}}_{3}^{-}$ consumption and loss from Samail Ophiolite aquifer fluids. We detected gene homologs for all major genes pertaining to denitrification (e.g., *nirS, nirK, norB*, and *nosZ*) (Philippot, 2002) in metagenomic sequencing of biomass from borehole fluids. High rates of denitrification have been reported in biological soil crusts in Oman associated with >300 μmol N/m^2^/h emissions of N_2_O (Abed et al., 2013). We detected N_2_O in nM concentrations (~ 5–176 nM) in sampled aquifer fluids (Table 5). However, many other N cycling processes could produce N_2_O, including the decomposition of intermediate hydroxylamine during NH_3_ oxidation, reduction of ${\text{NO}}_{2}^{-}$ by nitrifiers (nitrifier-denitrification), and reduction of ${\text{NO}}_{2}^{-}$ through reaction with ferrous iron (chemodenitrification) (Wankel et al., 2017). Furthermore, DNRA would be expected to occur at higher rates than denitrification at alkaline pH (Yoon et al., 2015) and where ${\text{NO}}_{3}^{-}$ is limited (Jørgensen, 1989; Kraft et al., 2014). In addition, the presence of assimilatory NR genes (e.g., *narB, nasA, nirA*) in aquifer fluids may indicate the potential for NR for assimilation into biomass instead of respiration (Moreno-Vivián et al., 1999); however, this process is unlikely to be the predominant ${\text{NO}}_{3}^{-}$ consuming process in Ca^2+^-OH^−^ type fluids where bioavailable N in the form of NH_3_ is abundant and biomass is low (5 × 10^5^ cells/mL) (Fones et al., 2019). The accompanying rise in ∑NH_3_ concentrations with a decrease in ${\text{NO}}_{3}^{-}$ concentrations and an increase in pH suggests that NR to ∑NH_3_ is proportionally a more significant process, at least in Ca^2+^-OH^−^ type fluids. However, additional measurements such as N_2_/Ar ratios (Vogel et al., 1981), ^15^N labeled assays, transcriptomics, and site-specific N_2_O isotopic analyses should be carried out in future investigations of this system to more definitively assess the potential for alternative biological NR processes.

### 4.4. The isotopic composition of ∑NH_3_ is highly variable in aquifer fluids

We observed a large range in δ^15^N of ∑NH_3_. We presume that δ^15^N_red_ is equivalent to the δ^15^N of ∑NH_3_ for all boreholes except NSHQ14 and WAB188, as sampled fluids from these wells exhibited comparable N_red_ and ∑NH_3_ concentrations (Table 3). The δ^15^N < −12‰ for ∑NH_3_ measured in BA1A contrasts with positive values measured in other boreholes. This could be related to the collection of fluids with the packer system (see Supplementary datasheet), which discretely sampled the lower borehole (100–400 m), as this sampled pool of ∑NH_3_ may reflect a pool that has undergone little oxidation by nitrifiers. The isotope effect for NH_3_ oxidation can be as large as −38‰ for bacterial nitrification (Mariotti et al., 1981; Yoshida, 1988; Casciotti et al., 2003), and thus the partial oxidation of NH_3_ in Ca^2+^-OH^−^ fluids could enrich the residual ∑NH_3_ pool, accounting in part for the higher δ^15^N values of ∑NH_3_ observed in other reacted fluids. In addition, there is a strong equilibrium isotope effect (−42.5‰) associated with ∑NH_3_ speciation, volatilization, and degassing (Li et al., 2012). If ammonia is lost through degassing, the residual pool of ${\text{NH}}_{4}^{+}$, and thus ∑NH_3_, should become increasingly enriched in ^15^N. Finally, some variability in the δ^15^N of ∑NH_3_ can be explained by groundwater age. The δ^15^N of ${\text{NO}}_{3}^{-}$ in atmospheric deposition has decreased by approximately 15‰ over the past century due to the Haber–Bosch effect of increased anthropogenic inputs from fertilizers (Yang and Gruber, 2016). Because Ca^2+^-OH^−^ fluids in Oman are pre–H-bomb (older than 1952), whereas Mg-${\text{HCO}}_{3}^{-}$ are estimated to be only 4–40 years old (Paukert Vankeuren et al., 2019), the δ^15^N composition of source ${\text{NO}}_{3}^{-}$ was not consistent across fluids.

### 4.5. Other potential sources of ∑NH_3_

Common sources of ∑NH_3_ in aquifer catchments such as remineralized organic matter, fertilizer, or wastewater (Kendall Carol, 1998) are unlikely for the Samail Ophiolite aquifer system. Due to the location of the Samail Ophiolite in the Omani desert, there is little agriculture or even human inhabitation in the catchments that supply the subsurface aquifer. Furthermore, N_red_ is within the standard error for measured ∑NH_3_ in most aquifer fluids. Dissolved organic N constitutes on average >80% of total N in anthropogenic runoff (Jani et al., 2020), thus we posit there is little contribution to reduced N from these sources where inorganic forms of N are predominant (see Supplementary datasheet for the discussion of samples where N_red_ >> ∑NH_3_).

A major potential source of ∑NH_3_ to Samail Ophiolite aquifers could be atmospheric deposition. Unfortunately, we did not acidify an aliquot of rainwater for analysis during the one rain event that coincided with our geochemical sampling, so the assessment of wet deposition was not possible. Whether through wet or dry deposition, atmospheric ∑NH_3_ could be effectively transported to the subsurface aquifer through rainfall. However, despite the apparent atmospheric source for a significant fraction of shallow aquifer ${\text{NO}}_{3}^{-}$, the same cannot be presumed for ∑NH_3_ because the atmospheric deposition of reduced nitrogen species (NH_x_) is not necessarily correlated with the magnitude of N-oxide deposition. Sources of oxidized and reduced N in the atmosphere are quite different, with NH_x_ primarily originating from agricultural pollution such as emissions from livestock and volatilization of fertilizers (Reis et al., 2009).

Another source of ∑NH_3_ to subsurface aquifers is biological N fixation. Although nitrogenase enzymes require high energetic costs to reduce N_2_ gas to ${\text{NH}}_{4}^{+}$ (Broda and Peschek, 1980), this process has been hypothesized to occur in some oligotrophic, rock-hosted environments such as the Henderson Mine and the serpentinite-hosted Lost City hydrothermal field (Sahl et al., 2008; Swanner and Templeton, 2011; Lang et al., 2013). While we did detect *nifD* gene homologs (which encode the catalytic site for the nitrogenase enzyme), their detection did not correlate with ∑NH_3_ concentrations. Alternatively, surficial ∑NH_3_ produced by diazotrophic biological soil crusts could be transferred to the subsurface *via* recharging rainfall. N fixation by soil crusts commonly occurs in arid ecosystems, including Oman (Abed et al., 2010, 2013); however, aquifers in the Samail Ophiolite are hosted in alluvium and mafic to ultramafic bedrock without soil cover (Dewandel et al., 2005), likely limiting fixed soil N contributions to the aquifer. Regardless of where N fixation may occur, the fractionation imparted by nitrogenases cannot fully account for the ~30‰ variation in or the lowest (−16.7‰) δ^15^N of ∑NH_3_ observed. N fixation with common molybdenum–based nitrogenase enzymes only imparts a small isotopic effect of −2 to +1‰ (Macko et al., 1987), and less efficient vanadium and iron–based alternative nitrogenase enzymes a fractionation of −6 to −8‰ (Zhang et al., 2014). Accordingly, while N fixation may contribute ∑NH_3_ to aquifer fluids, it seems unlikely as the primary source for the >100 μM ∑NH_3_ observed in some Ca^2+^-OH^−^ fluids.

Alternatively, rock-hosted N could be released during water–rock weathering reactions (Houlton et al., 2018). Although unaltered lithospheric peridotite has been found to have exceedingly low N concentrations (< 1 ppm) (Yokochi et al., 2009), the substitution of ${\text{NH}}_{4}^{+}$ for potassium, calcium, or sodium in silicate minerals occurs widely (Holloway and Dahlgren, 2002), particularly as a result of water/rock interaction. We measured up to 13.9 ppm bulk N in serpentinized peridotite, which is within the range of concentrations (~1–20 ppm) measured for altered ophiolitic glasses (Bebout et al., 2017) and serpentinized metaperidotites (Philippot et al., 2007; Halama et al., 2010), where N is presumed to occur as silicate-bound ${\text{NH}}_{4}^{+}$or trapped in fluid inclusions in sealed fractures produced during serpentinization reactions. Bulk δ^15^N of our measured peridotite samples varied from 3.5 to 6.7‰, similar to values observed in altered basalts and peridotites (Busigny et al., 2005; Philippot et al., 2007; Halama et al., 2010; Bebout et al., 2017) and consistent of a mantle signature with some incorporation of ${\text{NH}}_{4}^{+}$ from reacted fluids (Busigny and Bebout, 2013). It is unclear whether the incorporation of ${\text{NH}}_{4}^{+}$ is ongoing through modern water–rock interactions, or if this N could be released into the fluids during rock dissolution. Future studies should investigate the potential for leaching of ${\text{NH}}_{4}^{+}$ from serpentinized peridotite, especially because on a microscale, fluid compositions in porewaters could be differentially enriched in dissolved N through this mechanism.

### 4.6. Recycling of ∑NH_3_ in the near surface

${\text{NH}}_{4}^{+}$ produced *via* DNRA in reacted fluids, in combination with ∑NH_3_ from any aforementioned source, is recycled in the near surface through nitrification. Despite the highly reducing nature of Ca^2+^-OH^−^ fluids (Eh typically below −100 mV), evidence for aerobic nitrification can be observed in some boreholes such as WAB71 where the δ^18^O of ${\text{NO}}_{3}^{-}$ measured was < 10‰ across multiple years of sampling (Table 2). Nitrification is capable of proceeding at dissolved oxygen concentrations of 5–30 nM, or ~0.01% air saturation (Bristow et al., 2016), and thus NH_3_ oxidation could occur in the shallow aquifer where Ca^2+^-OH^−^ fluids come in contact with the atmosphere or mix with Mg-${\text{HCO}}_{3}^{-}$ type fluids.

Overall, the reduction of ${\text{NO}}_{3}^{-}$ to ${\text{NH}}_{4}^{+}$, as opposed to N_2_O or N_2_ gas, retains N in the subsurface aquifer, thus preventing N from acting as a limiting nutrient for biological growth. The oxidation of ∑NH_3_ produced by the reduction of atmospheric ${\text{NO}}_{3}^{-}$ through nitrification would then allow for further recycling of N in the subsurface, serpentinite-hosted aquifer ecosystem to sustain microbial growth even when the aquifers are not actively recharged. This continued cycling of atmospherically sourced ${\text{NO}}_{3}^{-}$ lends credence to the potential habitability of rock-hosted subsurface environments on other planetary bodies, such as Mars, where surficial inputs of ${\text{NO}}_{3}^{-}$ have been detected (Stern et al., 2017).

## 5. Conclusion

We employed dual N and O isotopic analysis of dissolved N species to probe the origin and subsequent cycling of ${\text{NO}}_{3}^{-}$ in Samail Ophiolite aquifer fluids since ${\text{NO}}_{3}^{-}$ is predicted to be a key electron acceptor for subsurface microbial life in terrestrial serpentinite ecosystems. ${\text{NO}}_{3}^{-}$ in all measured aquifer fluids was characterized by a positive Δ^17^O indicative of atmospheric deposition as a major source of oxidized N to the serpentinite-hosted aquifers. The Δ^17^O and δ^18^O of ${\text{NO}}_{3}^{-}$ in shallow aquifer fluids were consistent with simple mixing of ${\text{NO}}_{3}^{-}$ from atmospheric deposition with ${\text{NO}}_{3}^{-}$ produced *via* nitrification. However, ${\text{NO}}_{3}^{-}$ presumed to have formed through nitrification varied considerably in δ^15^N. In part, this is due to the ~30‰ variation of reactant ∑NH_3._ Concentrations of ∑NH_3_ increased concomitantly with a decrease in the concentration of ${\text{NO}}_{3}^{-}$ in more deeply sourced fluids, implying that NR could be a major source of ∑NH_3_ detected in Ca^2+^-OH^−^ fluids. The isotopic fractionation imparted by NR seemingly varied with ${\text{NO}}_{3}^{-}$ concentration, with greater fractionation (ε^15^ ~ −17.5‰) observed in shallow groundwaters, and less apparent fractionation (ε^15^ ~ −4.5‰) in deeper groundwaters where ${\text{NO}}_{3}^{-}$ concentrations were < 30 μM. This difference in isotope effect could be explained if ${\text{NO}}_{3}^{-}$ uptake becomes the rate-limiting step in NR in highly reacted fluids where ${\text{NO}}_{3}^{-}$ is scarce. The relationship between O and N isotopic fractionation (^18^ε/^15^ε) during ${\text{NO}}_{3}^{-}$ consumption was consistent with biological dissimilatory NR with a Nar reductase, although the possibility for abiotic reduction cannot be ruled out. Overall, the measured O and N isotopic compositions of ${\text{NO}}_{3}^{-}$ in Samail Ophiolite aquifer fluids are consistent with the recycling of atmospherically derived N through the initial reduction of meteoric ${\text{NO}}_{3}^{-}$ to ${\text{NH}}_{4}^{+}$ followed by partial (re)oxidation to ${\text{NO}}_{3}^{-}$ during nitrification in the near surface. This mode of biogeochemical cycling has major implications for the habitability of these aquifers, as the reduction of ${\text{NO}}_{3}^{-}$ to ${\text{NH}}_{4}^{+}$ retains N in the subsurface ecosystem.

## Data availability statement

Raw isotopic data presented in this study and source code used to produce the figures and data tables in this manuscript are available at https://github.com/KopfLab/OmanN_Cycling. Metagenomic sequence files are available from the MG-RAST database under accession numbers mgm4795805.3 to mgm4795809.3 and mgm4795811.3.

## Author contributions

AT, SK, JM, JS, and KR conceived the study. KR, DN, EK, JM, JS, and AT collected samples in the field. KR, DN, EK, CA, RDE, and SK analyzed samples and assisted in the data interpretation. KR wrote the manuscript. All authors critically revised the manuscript text and figures.
