# Supplementary material for: Subsurface biogeochemical cycling of nitrogen in the actively serpentinizing Samail Ophiolite, Oman
Source: Front Microbiol. 2023 Apr 21;14:1139633. doi: 10.3389/fmicb.2023.1139633 (PMC10160414; doi:10.3389/fmicb.2023.1139633)
Supplement: Supplementary file 5 [file Data_Sheet_1.docx]

Supplementary Material

Subsurface Biogeochemical Cycling of Nitrogen in the Actively Serpentinizing Samail Ophiolite, Oman

Kaitlin R. Rempfert*, Daniel B. Nothaft, Emily A. Kraus, Ciara K. Asamoto, R. Dave Evans, John R. Spear, Juerg M. Matter, Sebastian H. Kopf, Alexis S. Templeton^*^

*** Correspondence:** Kaitlin R. Rempfert: [kaitlin.rempfert@colorado.edu](mailto:kaitlin.rempfert@colorado.edu), Alexis S. Templeton: [alexis.templeton@colorado.edu](mailto:alexis.templeton@colorado.edu)

# Supplementary Figures

**Supplementary Figure 1.** Change of the nitrate δ^15^N and δ^18^O as a function of the natural logarithm of the fluid NO_3_^-^ concentration. The slopes of the linear trend lines correspond to the NO_3_^-^ N and O isotope effects of apparent nitrate consumption (ε), which vary from ~-4.5 (bold dashed line) to -17.5‰ (dotted line).

# Supplemental Text

## Estimates of Atmospheric Wet Deposition

NO_3_^-^ wet deposition (WD) fluxes were estimated by the product of NO_3_^-^ concentration [NO_3_^-^] in rainwater in mg/L and the annual rainfall (RF) and the annual rainfall (RF) amount in mm/year according to the following formula from (Keresztesi et al., 2020):

1. $WD \left( kg{ha}^{-1}{yr}^{-1} \right)=[NO_{3}^{-}]\left( mg L^{-1} \right)*\frac{RF}{100}$

We only captured one rainfall event in 2017 in which we measured NO_3_^-^ concentrations of 255 ± 20 µM. Since we did not collect samples throughout the entirety of the rainfall event, we could not calculate a volume weighted mean concentration for N-NO_3_^-^. However, using a concentration of 100 µM NO_3_^-^ reported by Weyhenmeyer (2002) for rainfall collected in the mountains of northern Oman as an additional constraint, and 80 mm of precipitation as the typical yearly rainfall, we estimate a wet atmospheric surface deposition of between 1.12 to 2.86 kg/ha/year of N-NO_3_^-^.

## δ^15^N of Nitrified NO_3_^-^

The δ^15^N of nitrified NO_3_^-^ (Supplemental Table 3) was variable, likely owing both to different isotopic compositions of reactant NH_4_^+^ as well as expressed isotope effects of nitrification. The N isotopic fractionation factor for NH_3_ oxidation, ε^15^_amo_, spans -14 to -38‰ in bacterial NH_3_ oxidizers (Casciotti et al., 2003; Mariotti et al., 1981; Yoshida, 1988) and -20 to -22‰ in archaea (Santoro and Casciotti, 2011). An additional inverse isotope effect of 0 to +35‰ is associated with NO_2_^-^ oxidation to NO_3_^-^ by the enzyme nitrite oxidoreductase (Casciotti, 2009). In many environments NH_3_ is completely consumed and NO_2_^-^ oxidation is tightly coupled to NH_3_ oxidation, thus imparting little to no fractionation from source NH_3_ δ^15^N (Casciotti & Buchwald, 2012; Granger & Wankel, 2016). In Samail Ophiolite aquifer fluids, we detect both ∑NH_3_ and NO_2_^-^ in most fluids, and so we assume some isotopic effect imparted at both steps, albeit small if concentrations are low and this reaction is thus diffusion-limited. We especially expect some isotopic fractionation during NH_3_ oxidation in Ca^2+^-OH^-^ fluids where ∑NH_3_ accumulates to concentrations as high as 114 µM, and only partial oxidation of this pool occurs. By removing the isotopic signature of the atmospheric NO_3_^-^ end member through calculation of the δ^15^N NO_3_^-^_biogeo,_ we infer a δ^15^N of ~ 3 to 5‰ for NO_3_^-^ that has a δ^18^O characteristic of nitrification (~ -0.4‰), however, the only fluids that demonstrated a near end-member nitrification signature were Mg^2+^-HCO_3_^-^ type fluids. Conversely, the δ^15^N of the presumed nitrified NO_3_^-^ in the Ca^2+^-OH^-^ borehole WAB71 was 13.4‰ for a corresponding δ^18^O of NO_3_^-^ of 0.8‰. While this 10‰ difference in the δ^15^N of nitrified NO_3_^-^ could be explained by differential rates (and thus isotopic expression) of NH_3_ and NO_2_^-^ oxidation, these processes are typically tightly coupled even in low oxygen environments such as OMZs (oxygen minimum zones) (Lau et al., 2019). Thus, we interpret these differences in the δ^15^N of nitrified NO_3_^-^ as arising from differences in the isotopic composition of reactant ammonia and the fractional extent of the NH_3_ that was oxidized.

## Unlikely Contribution of NO_3_^-^ by Annamox

We interpret the light δ^18^O NO_3_^-^ signature in aquifer fluids as the consequence of nitrification, not anaerobic ammonia oxidation (anammox), because gene homologs for hydrazine dehydrogenase (*hdh*) were only detected sparsely, and at low abundances, in the Samail Ophiolite metagenomes. This gene encodes the key enzyme in anammox for catalysis of hydrazine oxidation to dinitrogen gas during oxidation of NH_3_ with NO_2_^-^ as an electron acceptor (Kartal et al., 2007; Maalcke et al., 2016). It is important to note that anammox imparts a different isotopic effect than nitrification, with a combined inverse isotopic effect of NO_2_^-^ oxidation to NO_3_^-^ (incorporation of O atom from H_2_O and oxidation of NO_2_^-^) ranging from +1.5 to +12.1‰ (Kobayashi et al., 2019). For comparison, the combined isotope effect for aerobic nitrification is normal, ranging from -5 to -15‰ (Buchwald and Casciotti, 2010). While annamox can generate NO_3_^-^ with an oxygen isotopic composition far lighter than that of rainwater, the resultant NO_3_^-^ should have a δ^18^O value greater than the reactant NO_2_^-^. All measured NO_2_^-^ that was abundant enough for isotopic analysis demonstrated δ^18^O values >5‰ **(Table 5)**, and thus it is unlikely annamox could account for NO_3_^-^that has a δ^18^O signature similar to that of groundwater (i.e. <0‰), such as observed for the estimated δ^18^O of NO_3_^-^_biogeo_ in Mg^2+^-HCO_3_^-^ fluids. Still, we do not discount the possibility that anammox is also generating some of the observed NO_3_^-^ in this environment, especially because 16S rDNA sequences affiliated with the annamox bacterial order Brocadiales were previously detected in Samail ophiolite aquifer fluids (Rempfert et al., 2017).

## Role of NO_3_^-^ Concentration in Apparent Isotope Effects for NR

We hypothesize that differential apparent isotope effects for NO_3_^-^ consumption could arise if concentration modulates the rate-limiting step of NR. First, we assume that one process is imparting the apparent isotope effects during NO_3_^-^ consumption because the proportionality of N and O isotope fractionation (^18^ε/^15^ε) associated with NO_3_^-^  consumption in Samail Ophiolite aquifers was conserved. The approximated ^18^ε/^15^ε proportionality was ~1, which is consistent with biological NR (Bourbonnais et al., 2017; Casciotti & McIlvin, 2007; Casciotti et al., 2013; Gaye et al., 2013; Rafter et al., 2013). Second, we assume NR is multi-step, with a first step corresponding to uptake of NO_3_^-^ (in the case of biological NR, transport into the cell). Many studies have postulated that in multi-step consumption processes, the net isotope effect is controlled by the first irreversible step (Shearer et al., 1991; Kritee et al., 2012; Mariotti et al., 1981; O’Leary, 1981). As hypothesized by Kritee et al. (2012) for denitrification, at low (extracellular) concentrations of NO_3_^-^, NO_3_^-^  uptake could become the rate-limiting, irreversible step in cellular NR, thus controlling the magnitude of isotope fractionation. NO_3_^-^  uptake is thought to impart almost negligible fractionation (Shearer et al., 1991; Granger et al., 2010; Kritee et al., 2012), and thus could explain small isotopic effects for NR. In culturing experiments with denitrifying bacteria, Kritee et al. (2012) observed minimal fractionation during NR (10-15‰ compared to 20-30‰) when cells were grown at NO_3_^-^ concentrations below 35 µM. Concentrations of NO_3_^-^ in Ca^2+^-OH^-^ fluids were below 26 µM with the exception of borehole BA1A, supporting the theory that at low concentrations of NO_3_^-^, such as in most Ca^2+^-OH^-^ fluids, the isotope effect of NR could be reduced. If NH_4_^+^  is the product of NR, the difference in magnitude of this isotope effect in Mg^2+^-HCO_3_^-^ vs. Ca^2+^-OH^-^ fluids could largely explain the observed variation in isotopic composition of ∑NH_3_, especially if other processes that act to enrich ∑NH_3_ in Ca^2+^-OH^-^ fluids such as partial oxidation and degassing are co-occurring. Variable reactant NO_3_^-^  isotopic composition due to mixing, as indicated by the Leong et al. (2020) mixing model, would further introduce more deviation in product ∑NH_3_ isotopic composition.

## Hydrogeochemical Insights from Utilization of a Packer System

The only Mg^2+^-HCO_3_^-^ type fluid with ∑NH_3_ present at sufficient concentration for isotopic analysis was from borehole BA1A, the only borehole sampled with packers for collection of fluids from discrete depth intervals. The δ^15^N of N_red_ in both pumped intervals was notably light compared to all other measured samples, with a δ^15^N signature of -16.7‰ for N_red_ in the 55-66 m depth interval, and -12.9‰ in the 100-400 m depth interval. The concentration of N_red_ measured via mass balance after persulfate oxidation of these samples was consistent within error of the concentration of ∑NH_3_ measured through the salicylate colorimetric assay, indicating the δ^15^N of N_red_ should be representative of ∑NH_3_ at both intervals_._ While drilling of nearby borehole BA1B was ongoing during pumping of BA1A, we do not interpret this isotopically light ∑NH_3_ as contamination from drilling lubricant because fluorescent Invisible Blue® DayGlo particles used to trace the infiltration of drilling fluids ( Templeton et al., 2021) were not visible in sampled fluids in field by UV light. Additionally, the detection of even lighter nitrite (δ^15^N of NO_2_^-^: -17.9‰) in fluids collected two years prior in borehole NSHQ10 suggests an isotopically light source of NH_3_ is oxidized in Mg^2+^-HCO_3_^-^ fluids.

We hypothesize instead that utilization of the packer system allowed for the isolation of deeper groundwater without substantial dilution from the near-surface, oxygenated aquifer. Hydrologic testing conducted by Lods et al. (2020) at the time of our geochemical sampling indicated downflow in the BA1A borehole under ambient conditions (on the order of 1 L/min) in the depth interval of 22-59 meters. This downflow suggests near surface fluids are displaced to the lower aquifer from a highly transmissive aquifer situated at the contact between alluvium and dunite bedrock at a depth of 22-25 meters (Lods et al., 2020; Nothaft et al., 2021). We sampled the depth interval of 55-66 meters, which corresponded to an area of conductive fractures (41-75 m depth) (Lods et al., 2020). By packing off the upper 55 meters of the borehole, fluids sampled should originate primarily through horizontal flow through these conductive features. In contrast, the 100-400 m depth interval is interpreted to have primarily drawn more hyperalkaline and reduced waters from the surrounding formation from an aquifer situated at depths of 102-132 meters, although pumping tests indicated some combination of horizontal and vertical flow supplied these fluids (Lods et al., 2020; Nothaft et al., 2021). The reported predominance of sulfate-reducing taxa at both sampled depths (>20% relative abundance of rDNA amplicon sequence variants) by Nothaft et al. (2021) further supports the idea that sampled fluids are derived from depth, and not from the surface.

We interpret the coexistence of high NO_3_^-^ concentrations (>100 µM) with ∑NH_3_ concentrations >30 µM in BA1A borehole samples as the consequence of the reported downflow of shallow fluids into the lower aquifer, which could have effectively resupplied reacted, more deeply sourced Ca^2+^-OH^-^ type fluids with surficial NO_3_^-^. A near-surface origin of the NO_3_^-^ is also supported by the heavy (~6.9‰) Δ^17^O value of NO_3_^-^ in the 55-66-meter depth interval, indicating a large atmospheric contribution. At the 55-66-meter interval, downflow is more pronounced and thus the fluid composition resembles that of a Mg^2+^-HCO_3_^-^ fluid. However, the presence of ∑NH_3_ in fluids drawn from the fractured aquifer during pumping of the 55-66-meter depth interval suggest that O_2_ must be rapidly consumed in the upper tens of meters of the aquifer system to inhibit substantial nitrification. Amplicon sequencing conducted by Nothaft et al. (2021) of fluids from the 55-66-meter interval indicated the presence of Crenarchaeotal families Nitrosopumilaceae and Nitrososphaeraceae, two common NH_3_ oxidizers (Pester et al., 2012), suggesting the potential for this metabolism likely exists in these fluids. The rapid consumption of O_2_ in the upper meters of the aquifer would be consistent with the marked drop in Eh within the upper 40 meters of neighboring borehole BA1D where drilling-induced downflow of NO_3_^-^-rich fluids was not observed (Lods et al., 2020; Matter et al., 2018; Nothaft et al., 2021). The accumulation of some ∑NH_3_ at depth is not likely limited to Mg^2+^-HCO_3_^-^ type fluids in BA1A; we measured ∑NH_3_ in concentrations >5 µM in all boreholes sampled in 2018 via the salicylate colorimetric assay. However, without use of a packer system to isolate the lower aquifer systems, mixing with shallow alluvium fluids during pumping could dilute the concentration of ∑NH_3_ to below what would be required for confident isotopic analysis because this measurement is conducted via mass balance after persulfate oxidation.

## Isotope Effects for NH_3_ Degassing

There is a strong equilibrium isotope effect associated with ∑NH_3_ speciation and degassing. At the hyperalkaline pH observed for Ca^2+^-OH^-^ fluids, ∑NH_3_ should primarily be in the form of NH_3_ because the pKa for the disassociation of NH_4_^+^ to NH_3_ is ~ 9.25 at 25⁰ C (Olofsson, 1975). Li et al. (2012) estimated an isotopic equilibrium fractionation between NH4^+^ and aqueous NH_3_ of 42.5‰ for fluids at 33⁰ C through degassing experiments. Degassing under the Li et al. (2012) experimental conditions was rapid, with 50% of ∑NH_3_ degassed within two hours in experiments where NH4^+^ solutions were bubbled. However, the extent of degassing was highly dependent on not just pH, but also temperature and ambient fluid conditions (e,g, bubbled vs static). If any NH_3_ is lost through degassing, the residual pool of NH_4_^+^, and thus ∑NH_3,_ should become increasingly enriched (Li et al., 2012). This mechanism was invoked to explain the 50‰ δ^15^N signatures of bulk rock N in Archaean rocks of the Tumbiana Formation (Stüeken et al., 2015) and thus may commonly influence N isotope systematics in alkaline settings. Yet, much of the perceived isotope effect could be imparted during borehole pumping without a gas-tight sampler such as used with the packer system for BA1A. Because the heaviest δ^15^N of ∑NH_3_ (13.6‰) was observed in borehole WAB71 where the concentration of NH_3_ was greatest (114 µM), invoking any process that enriches the δ^15^N of ∑NH_3_ through removal of light NH_3_ to explain the heavy δ^15^N signatures of ∑NH_3_ in hyperalkaline fluids would imply substantial N loss from ammonia-rich fluids.

## Sources of N_red_ in wells NSHQ14 and WAB188

In wells NSHQ14 and WAB188, the concentration of N_red_ exceeded the measured concentration of ∑NH_3_. Well WAB188 is situated in gabbro; the hydraulic conductivity of gabbro-hosted aquifers is one to two orders of magnitude higher than that of peridotite-hosted aquifers (Dewandel et al., 2005) which could facilitate more effective transference of surface runoff to the subsurface. Additionally, both wells are located near the crust-mantle boundary where local faulting could promote fluid-mixing (Nicolas et al., 2000). We presume the greater contribution of dissolved organic N to the measured N_red_ pool reflects a greater influence of surface reduced N. While we cannot eliminate the possibility of agricultural or wastewater runoff as sources of reduced N at the surface, a predominance of organic N could also be explained by a greater influence of atmospheric dry deposition over precipitation in these samples. Wu et al. (2018) reported that dissolved organic N was the dominant species of N in aerosol particles, with >60% of N attributed to organic N.

# References

Bourbonnais, A., Letscher, R.T., Bange, H.W., Échevin, V., Larkum, J., Mohn, J., Yoshida, N., Altabet, M.A., 2017. N2O production and consumption from stable isotopic and concentration data in the Peruvian coastal upwelling system. Glob. Biogeochem. Cycles 31, 678–698. https://doi.org/10.1002/2016GB005567

Buchwald, C., Casciotti, K.L., 2010. Oxygen isotopic fractionation and exchange during bacterial nitrite oxidation. Limnol. Oceanogr. 55, 1064–1074. https://doi.org/10.4319/lo.2010.55.3.1064

Casciotti, K.L., 2009. Inverse kinetic isotope fractionation during bacterial nitrite oxidation. Geochim. Cosmochim. Acta 73, 2061–2076. https://doi.org/10.1016/j.gca.2008.12.022

Casciotti, K.L., Buchwald, C., 2012. Insights on the marine microbial nitrogen cycle from isotopic approaches to nitrification. Front. Microbiol. 3. https://doi.org/10.3389/fmicb.2012.00356

Casciotti, K.L., Buchwald, C., McIlvin, M., 2013. Implications of nitrate and nitrite isotopic measurements for the mechanisms of nitrogen cycling in the Peru oxygen deficient zone. Deep Sea Res. Part Oceanogr. Res. Pap. 80, 78–93. https://doi.org/10.1016/j.dsr.2013.05.017

Casciotti, K.L., McIlvin, M.R., 2007. Isotopic analyses of nitrate and nitrite from reference mixtures and application to Eastern Tropical North Pacific waters. Mar. Chem. 107, 184–201. https://doi.org/10.1016/j.marchem.2007.06.021

Casciotti, K.L., Sigman, D.M., Ward, B.B., 2003. Linking Diversity and Stable Isotope Fractionation in Ammonia-Oxidizing Bacteria. Geomicrobiol. J. 20, 335–353. https://doi.org/10.1080/01490450303895

Dewandel, B., Lachassagne, P., Boudier, F., Al-Hattali, S., Ladouche, B., Pinault, J.-L., Al-Suleimani, Z., 2005. A conceptual hydrogeological model of ophiolite hard-rock aquifers in Oman based on a multiscale and a multidisciplinary approach. Hydrogeol. J. 13, 708–726. https://doi.org/10.1007/s10040-005-0449-2

Gaye, B., Nagel, B., Dähnke, K., Rixen, T., Emeis, K.-C., 2013. Evidence of parallel denitrification and nitrite oxidation in the ODZ of the Arabian Sea from paired stable isotopes of nitrate and nitrite. Glob. Biogeochem. Cycles 27, 1059–1071. https://doi.org/10.1002/2011GB004115

Georgia, S., Schneider, J.D., Kohl, D.H., 1991. Separating the efflux and influx components of net nitrate uptake by Synechococcus R2 under steady-state conditions. Microbiology 137, 1179–1184. https://doi.org/10.1099/00221287-137-5-1179

Granger, J., Sigman, D.M., Rohde, M.M., Maldonado, M.T., Tortell, P.D., 2010. N and O isotope effects during nitrate assimilation by unicellular prokaryotic and eukaryotic plankton cultures. Geochim. Cosmochim. Acta 74, 1030–1040. https://doi.org/10.1016/j.gca.2009.10.044

Granger, J., Wankel, S.D., 2016. Isotopic overprinting of nitrification on denitrification as a ubiquitous and unifying feature of environmental nitrogen cycling. Proc. Natl. Acad. Sci. 113, E6391–E6400. https://doi.org/10.1073/pnas.1601383113

Kartal, B., Kuypers, M.M.M., Lavik, G., Schalk, J., Camp, H.J.M.O. den, Jetten, M.S.M., Strous, M., 2007. Anammox bacteria disguised as denitrifiers: nitrate reduction to dinitrogen gas via nitrite and ammonium. Environ. Microbiol. 9, 635–642. https://doi.org/10.1111/j.1462-2920.2006.01183.x

Keresztesi, Á., Nita, I.-A., Birsan, M.-V., Bodor, Z., Pernyeszi, T., Micheu, M.M., Szép, R., 2020. Assessing the variations in the chemical composition of rainwater and air masses using the zonal and meridional index. Atmospheric Res. 237, 104846. https://doi.org/10.1016/j.atmosres.2020.104846

Kobayashi, K., Makabe, A., Yano, M., Oshiki, M., Kindaichi, T., Casciotti, K.L., Okabe, S., 2019. Dual nitrogen and oxygen isotope fractionation during anaerobic ammonium oxidation by anammox bacteria. ISME J. 13, 2426–2436. https://doi.org/10.1038/s41396-019-0440-x

Kritee, K., Sigman, D.M., Granger, J., Ward, B.B., Jayakumar, A., Deutsch, C., 2012. Reduced isotope fractionation by denitrification under conditions relevant to the ocean. Geochim. Cosmochim. Acta 92, 243–259. https://doi.org/10.1016/j.gca.2012.05.020

Lau, E., Frame, C.H., Iv, E.J.N., Stewart, F.J., Dillard, Z.W., Lukich, D.P., Mihalik, N.E., Yauch, K.E., Kinker, M.A., Waychoff, S.L., 2019. Diversity and relative abundance of ammonia- and nitrite-oxidizing microorganisms in the offshore Namibian hypoxic zone. PLOS ONE 14, e0217136. https://doi.org/10.1371/journal.pone.0217136

Leong, J.A.M., Howells, A.E., Robinson, K.J., Cox, A., Debes, R.V., Fecteau, K., Prapaipong, P., Shock, E., 2020. Theoretical predictions vs environmental observations on serpentinization fluids: Lessons from the Samail ophiolite in Oman [WWW Document]. Earth Space Sci. Open Arch. https://doi.org/10.1002/essoar.10504642.1

Li, L., Lollar, B.S., Li, H., Wortmann, U.G., Lacrampe-Couloume, G., 2012. Ammonium stability and nitrogen isotope fractionations for NH4+–NH3(aq)–NH3(gas) systems at 20–70°C and pH of 2–13: Applications to habitability and nitrogen cycling in low-temperature hydrothermal systems. Geochim. Cosmochim. Acta 84, 280–296. https://doi.org/10.1016/j.gca.2012.01.040

Lods, G., Roubinet, D., Matter, J.M., LEPROVOST, R., Gouze, P., 2020. Groundwater flow characterization of an ophiolitic hard-rock aquifer from cross-borehole multi-level hydraulic experiments. J. Hydrol. 589, 125152. https://doi.org/10.1016/j.jhydrol.2020.125152

Maalcke, W.J., Reimann, J., de Vries, S., Butt, J.N., Dietl, A., Kip, N., Mersdorf, U., Barends, T.R.M., Jetten, M.S.M., Keltjens, J.T., Kartal, B., 2016. Characterization of Anammox Hydrazine Dehydrogenase, a Key N2-producing Enzyme in the Global Nitrogen Cycle. J. Biol. Chem. 291, 17077–17092. https://doi.org/10.1074/jbc.M116.735530

Mariotti, A., Germon, J.C., Hubert, P., Kaiser, P., Letolle, R., Tardieux, A., Tardieux, P., 1981. Experimental determination of nitrogen kinetic isotope fractionation: Some principles; illustration for the denitrification and nitrification processes. Plant Soil 62, 413–430. https://doi.org/10.1007/BF02374138

Matter, J.M., Pezard, P.A., Moe, K., Henry, G., Paris, J., Brun, L., Benchikh, A.M., Célérier, B., Lods, G., Al Shukaili, M., Al Amri, S., Al Qassabi, A., Kelemen, P.B., Teagle, D.A.H., Coggon, J.A., 2018. Advanced downhole hydrogeophysical logging during Oman Drilling Project Phase 2 - Correlation of hydraulic and fluid properties. AGU Fall Meet. Abstr. 12.

Nicolas, A., Boudier, F., Ildefonse, B., Ball, E., 2000. Accretion of Oman and United Arab Emirates ophiolite – Discussion of a new structural map. Mar. Geophys. Res. 21, 147–180. https://doi.org/10.1023/A:1026769727917

Nothaft, D., Templeton, A.S., Boyd, E., Matter, J., Stute, M., Vankeuren, A.N.P., 2021. Aqueous geochemical and microbial variation across discrete depth intervals in a peridotite aquifer assessed using a packer system in the Samail Ophiolite, Oman [WWW Document]. Earth Space Sci. Open Arch. https://doi.org/10.1002/essoar.10506402.2

O’Leary, M.H., 1981. Carbon isotope fractionation in plants. Phytochemistry 20, 553–567. https://doi.org/10.1016/0031-9422(81)85134-5

Olofsson, G., 1975. Thermodynamic quantities for the dissociation of the ammonium ion and for the ionization of aqueous ammonia over a wide temperature range. J. Chem. Thermodyn. 7, 507–514. https://doi.org/10.1016/0021-9614(75)90183-4

Pester, M., Rattei, T., Flechl, S., Gröngröft, A., Richter, A., Overmann, J., Reinhold-Hurek, B., Loy, A., Wagner, M., 2012. amoA-based consensus phylogeny of ammonia-oxidizing archaea and deep sequencing of amoA genes from soils of four different geographic regions. Environ. Microbiol. 14, 525–539. https://doi.org/10.1111/j.1462-2920.2011.02666.x

Rafter, P.A., DiFiore, P.J., Sigman, D.M., 2013. Coupled nitrate nitrogen and oxygen isotopes and organic matter remineralization in the Southern and Pacific Oceans. J. Geophys. Res. Oceans 118, 4781–4794. https://doi.org/10.1002/jgrc.20316

Rempfert, K.R., Miller, H.M., Bompard, N., Nothaft, D., Matter, J.M., Kelemen, P., Fierer, N., Templeton, A.S., 2017. Geological and Geochemical Controls on Subsurface Microbial Life in the Samail Ophiolite, Oman. Front. Microbiol. 8. https://doi.org/10.3389/fmicb.2017.00056

Santoro, A.E., Casciotti, K.L., 2011. Enrichment and characterization of ammonia-oxidizing archaea from the open ocean: phylogeny, physiology and stable isotope fractionation. ISME J. 5, 1796–1808. https://doi.org/10.1038/ismej.2011.58

Stüeken, E.E., Buick, R., Schauer, A.J., 2015. Nitrogen isotope evidence for alkaline lakes on late Archean continents. Earth Planet. Sci. Lett. 411, 1–10. https://doi.org/10.1016/j.epsl.2014.11.037

Templeton, A.S., Ellison, E.T., Glombitza, C., Morono, Y., Rempfert, K.R., Hoehler, T., Zeigler, S.D., Kraus, E., Spear, J., Nothaft, D., Fones, E.M., Boyd, E., Munro-Ehrlich, M., Mayhew, L., Cardace, D., Matter, J., Kelemen, P.B., Party, the O.D.P.S., 2021. Accessing the subsurface biosphere within rocks undergoing active low-temperature serpentinization in the Samail ophiolite (Oman Drilling Project). Earth Space Sci. Open Arch. https://doi.org/10.1002/essoar.10506393.1

Weyhenmeyer, C.E., 2002. Groundwater Evolution in an Arid Coastal Region of the Sultanate of Oman based on Geochemical and Isotopic Tracers, in: Stober, I., Bucher, K. (Eds.), Water-Rock Interaction, Water Science and Technology Library. Springer Netherlands, Dordrecht, pp. 1–38. https://doi.org/10.1007/978-94-010-0438-1_1

Wu, Y., Zhang, J., Liu, S., Jiang, Z., Huang, X., 2018. Aerosol concentrations and atmospheric dry deposition fluxes of nutrients over Daya Bay, South China Sea. Mar. Pollut. Bull. 128, 106–114. https://doi.org/10.1016/j.marpolbul.2018.01.019

Yoshida, N., 1988. 15 N-depleted N 2 O as a product of nitrification. Nature 335, 528–529. https://doi.org/10.1038/335528a0
